# Investigating the initial steps of auricin biosynthesis using synthetic biology

**DOI:** 10.1186/s13568-023-01591-2

**Published:** 2023-08-08

**Authors:** Dominika Csolleiova, Rachel Javorova, Renata Novakova, Lubomira Feckova, Maria Matulova, Filip Opaterny, Bronislava Rezuchova, Beatrica Sevcikova, Jan Kormanec

**Affiliations:** 1 Institute of Molecular Biology, Slovak Academy of Sciences, Dubravska Cesta 21, 845 51 Bratislava, Slovak Republic; 2 Institute of Chemistry, Slovak Academy of Sciences, 845 38 Bratislava, Slovak Republic

**Keywords:** Antibiotics, Auricin, Biosynthetic gene cluster, Griseusin, Synthetic biology, *Streptomyces*

## Abstract

*Streptomyces lavendulae* subsp. *lavendulae* CCM 3239 (formerly *Streptomyces aureofaciens* CCM 3239) contains a type II polyketide synthase (PKS) biosynthetic gene cluster (BGC) *aur1* whose genes were highly similar to angucycline BGCs. However, its product auricin is structurally different from all known angucyclines. It contains a spiroketal pyranonaphthoquinone aglycone similar to griseusins and is modified with d-forosamine. Here, we describe the characterization of the initial steps in auricin biosynthesis using a synthetic-biology-based approach. We have created a plasmid system based on the strong kasOp* promoter, RBS and phage PhiBT1-based integration vector, where each gene in the artificial operon can be easily replaced by another gene using unique restriction sites surrounding each gene in the operon. The system was validated with the initial landomycin biosynthetic genes *lanABCFDLE*, leading to the production of rabelomycin after its integration into *Streptomyces coelicolor* M1146. However, the *aur1DEFCGHA* homologous genes from the auricin *aur1* BGC failed to produce rabelomycin in this system. The cause of this failure was inactive *aur1DE* genes encoding ketosynthases α and β (KSα, KSβ). Their replacement with homologous *aur2AB* genes from the adjacent *aur2* BGC resulted in rabelomycin production that was even higher after the insertion of two genes from the *aur1* BGC, *aur1L* encoding 4-phosphopantetheinyl transferase (PPTase) and *aur1M* encoding malonyl-CoA:ACP transacylase (MCAT), suggesting that Aur1L PPTase is essential for the activation of the acyl carrier protein Aur1F. These results suggest an interesting communication of two BGCs, *aur1* and *aur2*, in the biosynthesis of the initial structure of auricin aglycone.

**Supplementary Information:**

The online version contains supplementary material available at 10.1186/s13568-023-01591-2.

## Key points

- A new synthetic biology-based system for investigating antibiotic biosynthesis.
- Efficient rabelomycin production using the initial landomycin biosynthetic genes.
- Linking of two BGCs in the initial steps of auricin biosynthesis.

**Supplementary Information:**

The online version contains supplementary material available at 10.1186/s13568-023-01591-2.

## Introduction

Gram-positive bacteria of the genus *Streptomyces* are characterised by the production of a considerable number of secondary metabolites, many of them with various biological activities (Hopwood 2007). A large group of these secondary metabolites belong to aromatic polyketides, which are synthesized by a type II polyketide synthase (PKS). It is a multienzyme complex consisting of a minimal PKS, which contains α subunit and β subunit of ketoacyl synthase (KSα and KSβ) and an acyl carrier protein (ACP), producing a linear basic polyketide whose length is determined by a KSβ. Associated ketoreductases (KRs), aromatases (AROs), and cyclases (CYCs) modify this nascent chain to form an aromatic cyclic polyketide, which is subsequently modified by tailoring enzymes (such as oxygenases, reductases, methyltransferases, glycosyltransferases) to the final biologically active polyketide. Although a large repertoire of aromatic polyketides has been identified, they all belong to just a few common structural types, which include anthracyclines, tetracyclines, tetracenomycines, pyranonaphthoquinones, angucyclines, aureolic acids, and pradimycin-type polyphenols (Hertweck et al. 2007). Angucyclines represent the dominant group of these aromatic polyketides and exhibit a wide spectrum of biological activities. Their biosynthesis differs from other classes of aromatic polyketides by the action of a specific CYC, which closes the fourth ring in an angular orientation to form a common intermediate UWM6 (Kharel et al. 2012b).

In general, the biosynthetic genes for secondary metabolites in streptomycetes are physically grouped together with regulatory and resistance genes in so-called biosynthetic gene clusters (BGCs) located on large linear chromosomes or in some cases on large linear plasmids (Hopwood 2007). In the past, *Streptomyces* strains were thought to produce one or at best several different bioactive secondary metabolites. However, the analysis of more than a thousand of genomes from different *Streptomyces* strains showed a large number of BGCs for secondary metabolites of various groups (39 BGCs per genome on average). However, in laboratory conditions, most of them are silent (Belknap et al. 2020). To activate these silent BGCs, various approaches have been used, such as insertions of strong promoters into BGCs, overproductions or deletions of regulatory genes, and their transfer into optimal heterologous *Streptomyces* hosts allowing their expression (Baltz 2016; Kormanec et al. 2020). Another promising approach is synthetic biology, which in recent years has seen rapid progress in biotechnological applications. This approach is based on dividing BGCs into separate blocks of specific biosynthetic genes, in which native regulatory elements are replaced by strong promoters and ribosome binding sites (RBSs) to enable high and coordinated gene expression of these biosynthetic genes. Such artificial BGCs can be introduced into a heterologous *Streptomyces* strain with a simple secondary metabolism profile, allowing easy identification and purification of secondary metabolites (Lee et al. 2019; Palazzotto et al. 2019).

In our model strain *Streptomyces aureofaciens* CCM 3239, we identified a partial *aur1* BGC whose genes showed the highest similarity to angucycline BGCs, which was responsible for the production of antibiotic auricin (Novakova et al. 2002). Interestingly, the *aur1* BGC is located on the large linear plasmid pSA3239 (Novakova et al. 2013). However, sequence analysis of the whole genome of this strain (GenBank Acc. No. CP024985) showed that this strain was incorrectly assigned by the Czech Collection of Microorganisms (CCM) and is actually *Streptomyces lavendulae* subsp. *lavendulae* CCM 3239 (Busche et al. 2018). Sequencing of the complete *aur1* BGC, including its surroundings, showed its unusual organization. It consists of the *aur1A-aur1N* core region (Additional file 1: Figure S1) containing the genes that were homologous to the angucycline genes (Additional file 1: Figure S2) and several auricin-specific tailoring biosynthetic genes (including D-forosamine biosynthetic genes) were scattered up to 30 kb from this core region (Bekeova et al. 2016; Mingyar et al. 2018). In addition, the *aur1* BGC was disrupted by biosynthetic genes for another unrelated secondary metabolite, the blue pigment indigoidine (Novakova et al. 2010b). Next to the *aur1* BGC, there is another partial *aur2* BGC containing type II PKS genes similar to pyranonaphthoquinone or aureolic acid biosynthetic genes (Additional file 1: Figure S1) (Matulova et al. 2019; Mingyar et al. 2018).

Auricin was produced at very small quantities, which prevented its structural elucidation for many years. A detailed study showed an interesting pattern of its production. Auricin is produced in a narrow interval after entering the stationary phase, after which it is degraded due to its instability at the high pH values reached in the stationary phase (Kutas et al. 2013). This unusual pattern is also a result of its complex regulation with several transcriptional regulators (Kormanec et al. 2014; Bekeova et al. 2016; Novakova et al. 2022b). Purified auricin from this transient growth phase was stable in various solvents, allowing its purification and structure elucidation. Interestingly, these studies showed that auricin is structurally different from all known angucyclines. It contains a spiroketal pyranonaphthoquinone aglycone similar to griseusins and is modified with d-forosamine (Additional file 1: Figure S3) (Matulova et al. 2019).

The aim of the present study was to establish and optimize a synthetic biology expression system to characterize the initial steps of auricin biosynthesis, as there are no published data on the biosynthesis of spiroketal griseusins and the deletion of several auricin biosynthetic genes resulted in the loss of auricin without any stable intermediate (J. Kormanec, unpublished results).

## Materials and methods

### Bacterial strains, plasmids, and culture conditions

Plasmids and strains used in this study are listed in Additional file 1: Table S1. Conditions for growth and sporulation of *S. lavendulae* subsp. *lavendulae* CCM 3239 wild type (WT) and mutant strains are described in Matulova et al. (2019) and those for *S. coelicolor* strains in Kieser et al. (2000). Solid Bennet medium (Matulova et al. 2019) was used to propagate *S. lavendulae* subsp. *lavendulae* CCM 3239 and SFM medium (Kieser et al. 2000) for *S. coelicolor* strain. *Streptomyces* strains were grown in liquid corn steep liquor-sucrose (CSLS) inoculation medium (McCormick et al. 1959), CGGM medium (Kormanec et al. 1992), and Bennet medium (Matulova et al. 2019). *Escherichia coli* strains were grown in Luria–Bertani (LB) medium and their transformation is described in Ausubel et al. (1995). For standard cloning experiments, *E. coli* DH5α (ThermoFisher Scientific, Waltham, MA, USA) was used as the host. For transfer of plasmids by conjugation from *E. coli* into *Streptomyces* strains, the non-methylating *E. coli* ET12567/pUZ8002 was used (Kieser et al. 2000). When necessary, media were supplemented with 50 μg/ml kanamycin (Kan) (Cat. No. 26899.03, SERVA, Heidelberg, Germany), 25 μg/ml chloramphenicol (Clm) (Cat. No. C0378, Sigma-Aldrich, Darmstadt, Germany), 50 μg/ml apramycin (Apr) (Cat. No. A2024, Sigma-Aldrich, Darmstadt, Germany), 100 μg/ml ampicillin (Amp) (Cat. No. A9518, Sigma-Aldrich, Darmstadt, Germany), and 20 μg/ml nalidixic acid (NA) (Cat. No. N8878, Sigma-Aldrich, Darmstadt, Germany).

### Recombinant DNA techniques

Standard procedures for gene manipulation in *E. coli* were done as described by Ausubel et al. (1995). Chromosomal DNA from *Streptomyces* strains was prepared after cultivation for 19 h in CGGM medium as described by Kormanec et al. (2021). Correct gene deletions in *Streptomyces* chromosomes were verified by Southern blot hybridization analysis (Ausubel et al. 1995) using a Hybond N membrane (Roche, Manheim, Germany) with digoxigenin (DIG)-labelled probes according to the standard DIG protocol with a chemiluminescent detection kit (Roche, Manheim, Germany). The DIG-probes were prepared by PCR amplification with two suitable primers, *S. lavendulae* subsp. *lavendulae* CCM 3239 chromosomal DNA as a template, and PCR DIG Probe Synthesis kit (Roche, Manheim, Germany). Probe 1 (Additional file 1: Figure S4) was a 728-bp fragment containing the *aur1B* gene region, amplified with primers Aur1Bdir and Aur1Brev (Additional file 1: Table S2), and probe 2 (Additional file 1: Figure S5) was an 1122-bp fragment containing the *aur2BCD* region, amplified with primers Aur2Cdir and Aur2Crev (Additional file 1: Table S2).

### Construction of pErmEp-lanABCFDLE and pKasOp-lanABCFDLE

**Fig. 1:**
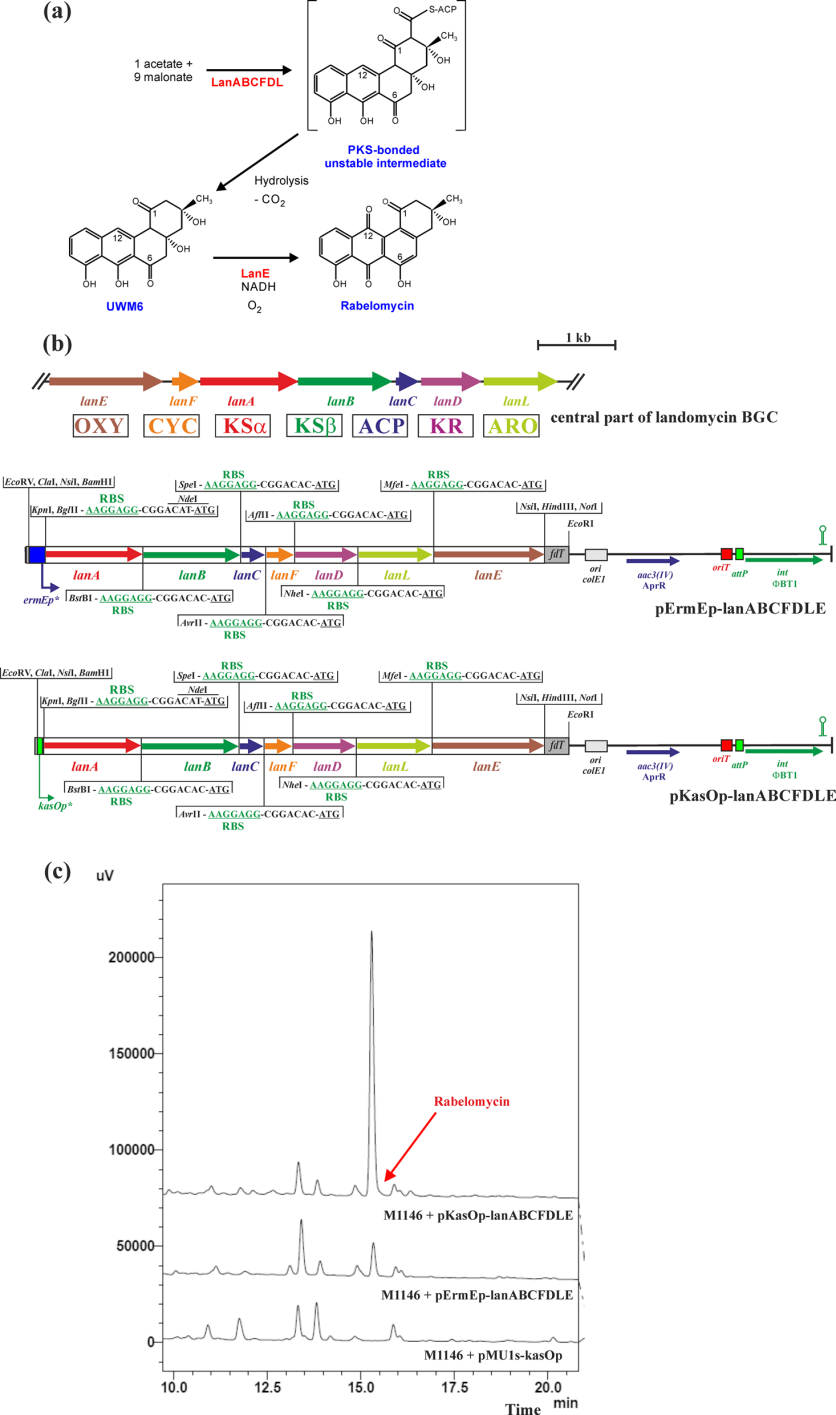
**a** Pathway of the initial steps in the biosynthesis of landomycin to form the shunt product rabelomycin (Kharel et al. 2012a). **b** Schemes of the integration plasmids pErmEp-lanABCFDLE and pKasOp-lanABCFDLE, which contain an artificial *lanABCFDL* operon under the control of the ermEp* or kasOp* promoter. Wild-type *lanEFABCDL* central region encoding oxygenase (OXY), angucycline-specific cyclase (CYC), ketosynthase α (KSα), ketosynthase β (KSβ), acyl carrier protein (ACP), ketoreductase (KR), and aromatase (ARO), is shown above the diagrams. Sequences upstream of individual genes are shown with the ribosome binding site (RBS) from pMU1s* (Craney et al. 2007) in green and the ATG codon underlined. The plasmid backbone contains the *fdT* terminator (dark grey box), the *E. coli* origin of replication *ColE1* (light grey box), the AprR *aac3(IV)* gene (blue arrow), the *oriT* origin of transfer (red bar), and the *phiBT1int* integrase gene (green arrow) together with the *attP* site (green bar) from plasmid pMU1s*. Relevant restriction sites are shown. **c** HPLC analysis of metabolites produced by representative clones from the indicated *S. coelicolor* M1146 strains containing integrated plasmids after growth in liquid Bennet medium for 3 days. The rabelomycin peak is indicated by an arrow

First, all seven initial landomycin biosynthetic genes, *lanA, lanB, lanC, lanF, lanD, lanL, lanE,* from the landomycin BGC of *S. cyanogenus* S136 (Westrich et al. 1999) were amplified by PCR with high-fidelity Q5 DNA polymerase (New England Biolabs, San Diego, CA, USA), cosmid H2-26 (Westrich et al. 1999) as a template, and selected primers LanXdir and LanXrev (Additional file 1: Table S2). The primer LanXdir introduced the *Xho*I cloning site, a unique restriction site, and a strong ribosome binding site (RBS) from pMU1s* (Craney et al. 2007) upstream of the respective gene; the primer LanXrev introduced another unique restriction site and another cloning site (*Hin*dIII, or *Not*I for *lanA*) downstream of the respective gene. DNA fragments were digested with *Xho*I and *Hin*dIII (or *Xho*I and *Not*I for *lanA*) (all from New England Biolabs, San Diego, CA, USA) and ligated into plasmid pBluescript II SK + digested with the same restriction enzymes, resulting in pBS-lanA, pBS-lanB, pBS-lanC, pBS-lanF, pBS-lanD, pBS-lanL, pBS-lanE (Additional file 1: Figure S6). The correctness of the recombinant plasmids was verified by nucleotide sequencing using forward and reverse primers -47 and -48 (Additional file 1: Table S2). The resulting recombinant plasmids were digested with the appropriate unique restriction enzymes and the individual genes together with RBS were sequentially inserted into the artificial synthetic operon *lanABCFDLE* using the cloning strategy shown in Additional file 1: Figure S6, resulting in plasmid pBS-lanABCFDLE. Subsequently, the 6.4-kb *Nde*I-*Not*I DNA fragment from pBS-lanABCFDLE was cloned into both expression vectors pMU1s-ermEp (Csolleiova et al. 2021) and pMU1s-kasOp (Novakova et al. 2022a, b) digested with the same restriction enzymes, resulting in the final recombinant integration plasmids pErmEp-lanABCFDLE and pKasOp-lanABCFDLE (Fig. 1b).

### Construction of pKasOp-aur1DEFCGHA, pKasOp-aur1DEFlanFDLE and pKasOp-lanABCaur1CGHA

**Fig. 2:**
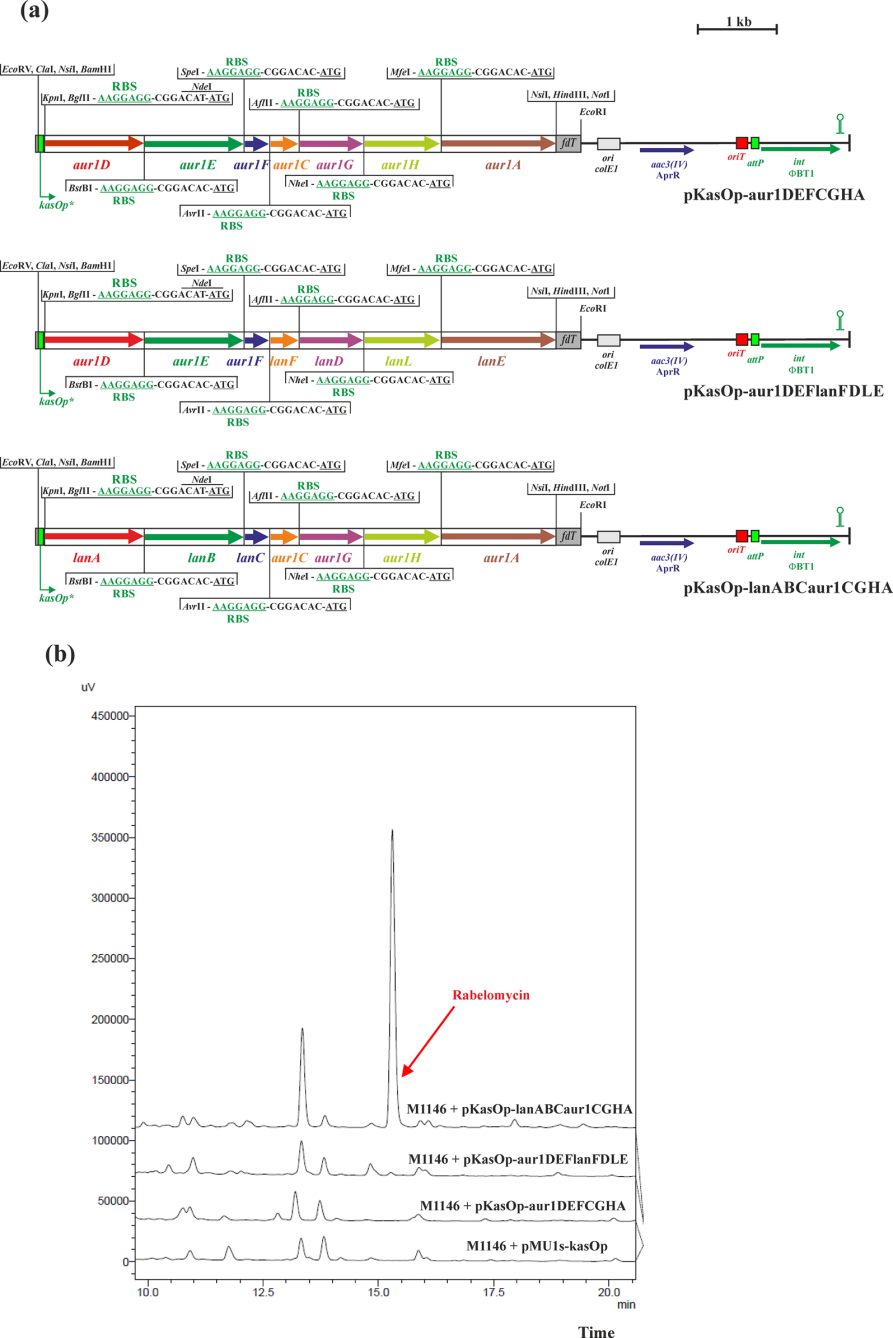
**a** Schemes of the integration plasmid pKasOp-aur1DEFCGHA, which contain an artificial *aur1DEFCGHA* operon under the control of the kasOp* promoter, and plasmids pKasOp-aur1DEFlanFDLE and pKasOp-lanABCaur1CGHA, containing mixed auricin/landomycin artificial operons. Sequences upstream of individual genes are shown with RBS from pMU1s* (Craney et al. 2007) in green and the ATG codon underlined. Description of the plasmid backbone is in the legend to Fig. 1. Relevant restriction sites are shown. **b** HPLC analysis of metabolites produced by representative clones from the indicated *S. coelicolor* M1146 strains containing integrated plasmids after growth in liquid Bennet medium for 3 days. The rabelomycin peak is indicated by an arrow

Using the identical cloning strategy as described above for landomycin biosynthetic genes, all seven homologous genes *aur1D, aur1E, aur1F, aur1C, aur1G, aur1H, aur1A,* from the core auricin BGC *aur1* of *S. lavendulae* subsp. *lavendulae* CCM 3239 (Novakova et al. 2002; Kormanec et al. 2014) (Additional file 1: Figure S1) were similarly PCR amplified with *S. lavendulae* subsp. *lavendulae* CCM 3239 chromosomal DNA as a template and selected primers Aur1Xdir and Aur1Xrev (Additional file 1: Table S2). The primer Aur1Xdir introduced the *Xho*I cloning site, a unique restriction site, and RBS from pMU1s* upstream of the respective auricin biosynthetic gene; the primer Aur1Xrev introduced another unique restriction site and another cloning site (*Hin*dIII, or *Not*I for *aur1D*) downstream of the respective gene. DNA fragments were digested with *Xho*I and *Hin*dIII (or *Xho*I and *Not*I for *aur1D*) and ligated into plasmid pBluescript II SK + digested with the same restriction enzymes, resulting in pBS-aur1D, pBS-aur1E, pBS-aur1F, pBS-aur1C, pBS-aur1G, pBS-aur1H, pBS-aur1A. The correctness of the recombinant plasmids was verified by nucleotide sequencing using forward and reverse primers -47 and -48 (Additional file 1: Table S2). The resulting recombinant plasmids were digested with the appropriate unique restriction enzymes and the individual genes together with RBS were sequentially inserted into the artificial synthetic operon *aur1DEFCGHA* using the identical cloning strategy shown for landomycin in Additional file 1: Figure S6, resulting in plasmid pBS-aur1DEFCGHA. Subsequently, the 6.4-kb *Nde*I-*Not*I DNA fragment from pBS-aur1DEFCGHA was cloned into the expression vector pMU1s-kasOp digested with the same restriction enzymes, resulting in the final recombinant integration plasmid pKasOp-aur1DEFCGHA (Fig. 2a).

To prepare mixed auricin/landomycin artificial operons, a 2.9-kb *Nde*I-*Avr*II DNA fragment from pBS-aur1DEFCGHA (containing the *aur1DEF* operon) was cloned into pKasOp-lanABCFDLE (replacing the *lanABC* genes) digested with the same restriction enzymes, resulting in pKasOp-aur1DEFlanFDLE (Fig. 2a). Similarly, a 3.2-kb *Avr*II-*Hin*dIII DNA fragment from pBS-aur1DEFCGHA (containing the *aur1CGHA* operon) was cloned into pKasOp-lanABCFDLE (replacing the *lanFDLE* genes) digested with the same restriction enzymes, resulting in pKasOp-lanABCaur1CGHA (Fig. 2a).

### Construction of pKasOp-aur2ABtlanCFDLE, pKasOp-aur1DEtlanCFDLE, pKasOp-aur2ABtFCGHA, and pKasOp-aur1DEtFCGHA

To prepare additional auricin/landomycin mixed artificial operons with two additional *aur2A* and *aur2B* genes, encoding KSα and KSβ from the *aur2* BGC located in a distant region from the *aur1* core BGC (Additional file 1: Figure S1), both genes were PCR amplified as the *aur2AB* operon with *S. lavendulae* subsp. *lavendulae* CCM 3239 chromosomal DNA as a template and primers Aur2Adir and Aur2Brev (Additional file 1: Table S2). The primers introduced the *Xho*I cloning site and RBS from pMU1s* upstream of the *aur2A* gene and a unique *Avr*II restriction site, another *Hin*dIII cloning site downstream of the *aur2B* gene. A 2.5-kb DNA fragment was digested with *Xho*I and *Hin*dIII and ligated into plasmid pBluescript II SK + digested with the same restriction enzymes, resulting in pBS-aur2ABt. Similarly, both *aur1D* and *aur1E* genes were PCR amplified as the *aur1DE* operon with *S. lavendulae* subsp. *lavendulae* CCM 3239 chromosomal DNA as a template and primers Aur1Ddir and Aur1Erev (Additional file 1: Table S2), which introduced the *Xho*I cloning site and RBS from pMU1s* upstream of the *aur1D* gene and a unique *Spe*I restriction site, another *Hin*dIII cloning site downstream of the *aur1E* gene. A 2.5-kb DNA fragment was digested with *Xho*I and *Hin*dIII and ligated into plasmid pBluescript II SK + digested with the same restriction enzymes, resulting in pBS-aur1DEt. The correctness of both recombinant plasmids was verified by nucleotide sequencing using forward and reverse primers -47 and -48 (Additional file 1: Table S2).

**Fig. 3:**
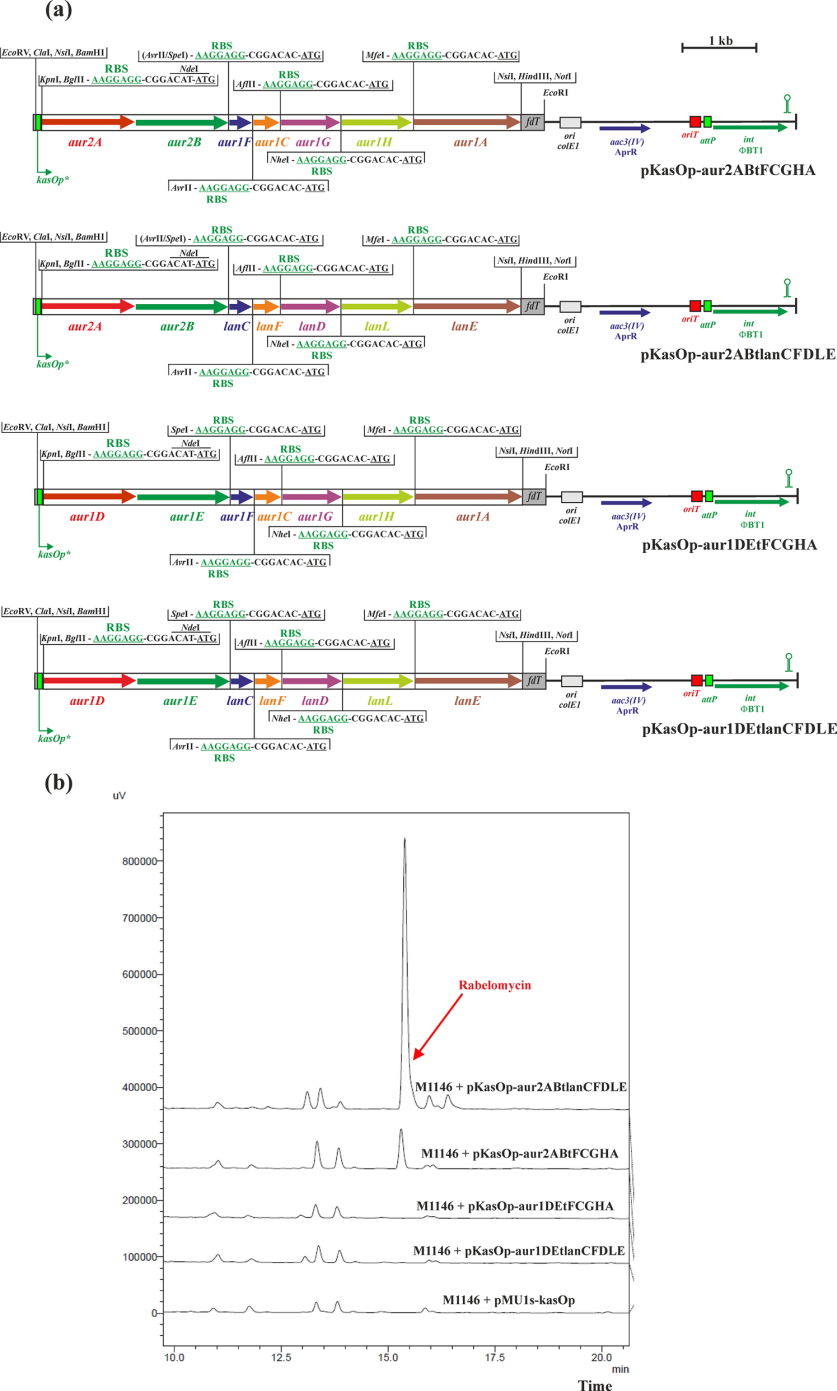
**a** Schemes of the integration plasmids pKasOp-aur2ABtFCGHA, pKasOp-aur2ABtlanCFDLE, pKasOp-aur1DEtFCGHA, and pKasOp-aur1DEtlanCFDLE, containing mixed auricin/landomycin artificial operons. Sequences upstream of individual genes are shown with RBS from pMU1s* (Craney et al. 2007) in green and the ATG codon underlined. Description of the plasmid backbone is in the legend to Fig. 1. Relevant restriction sites are shown. **b** HPLC analysis of metabolites produced by representative clones from the indicated *S. coelicolor* M1146 strains containing integrated plasmids after growth in liquid Bennet medium for 3 days. The rabelomycin peak is indicated by an arrow

A 2.5-kb *Nde*I-*Avr*II DNA fragment from pBS-aur2ABt was cloned into pKasOp-lanABCFDLE (replacing the *lanAB* genes) and into pKasOp-aur1DEFCGHA (replacing the *aur1DE* genes) digested with the same restriction enzymes, resulting in pKasOp-aur2ABtlanCFDLE and pKasOp-aur2ABtFCGHA (Fig. 3a). Similarly, a 2.5-kb *Nde*I-*Spe*I DNA fragment from pBS-aur1DEt was cloned into pKasOp-lanABCFDLE (replacing the *lanAB* genes) and pKasOp-aur1DEFCGHA (replacing separated *aur1DE* genes) digested with the same restriction enzymes, resulting in pKasOp-aur1DEtlanCFDLE and pKasOp-aur1DEtFCGHA (Fig. 3a).

### Construction of pKasOp-aur2ABtFCGHALM and pKasOp-aur1DEtFCGHALM

**Fig. 4:**
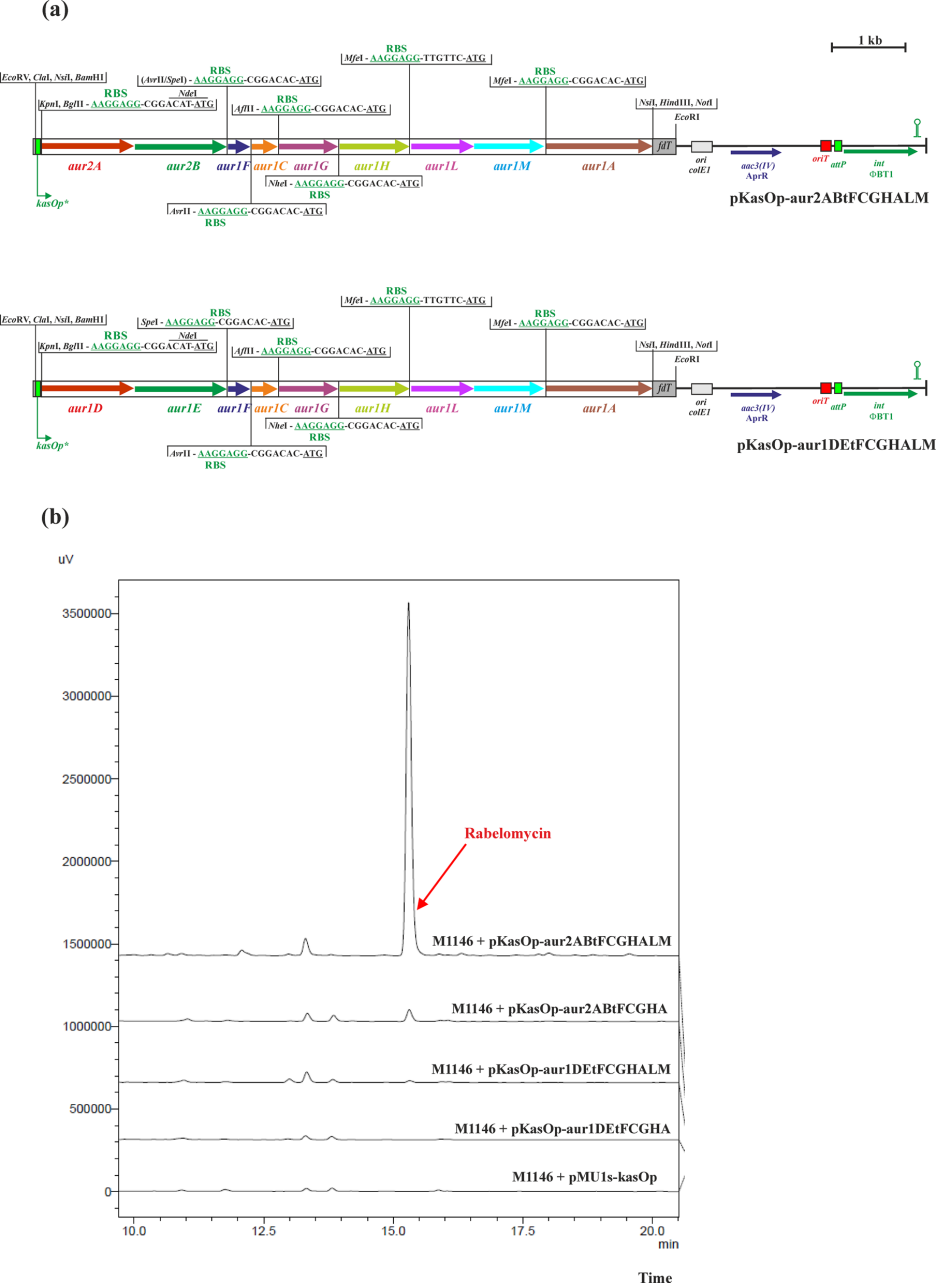
**a** Schemes of the integration plasmids pKasOp-aur2ABtFCGHALM and pKasOp-aur1DEtFCGHALM, containing mixed auricin artificial operons. Sequences upstream of individual genes are shown with RBS from pMU1s* (Craney et al. 2007) in green and the ATG codon underlined. Description of the plasmid backbone is in the legend to Fig. 1. Relevant restriction sites are shown. **b** HPLC analysis of metabolites produced by representative clones from the indicated *S. coelicolor* M1146 strains containing integrated plasmids after growth in liquid Bennet medium for 3 days. The rabelomycin peak is indicated by an arrow

Two genes, *aur1L* and *aur1M*, encoding 4-phosphopantetheinyl transferase (PPTase) and malonyl-CoA:ACP transacylase (MCAT), from the core auricin *aur1* BGC (Additional file 1: Figure S1) were PCR amplified as the *aur1LM* operon with *S. lavendulae* subsp. *lavendulae* CCM 3239 chromosomal DNA as a template and primers Aur1Ldir and Aur1Mrev (Additional file 1: Table S2). The Aur1Ldir primer introduced the *Xho*I cloning site, the *Mfe*I restriction site, and a strong RBS upstream of the *aur1L* gene; the Aur1Mrev primer introduced the *Mfe*I restriction site and another *Hin*dIII cloning site downstream of the *aur1M* gene. A 1.8-kb DNA fragment was digested with *Xho*I and *Hin*dIII and ligated into plasmid pBluescript II SK + digested with the same restriction enzymes, resulting in pBS-aur1LMt. The correctness of the recombinant plasmids was verified by nucleotide sequencing using forward and reverse primers -47 and -48 (Additional file 1: Table S2). A 1.8-kb *MfeI* DNA fragment from pBS-aur1LMt was cloned into pKasOp-aur2ABtFCGHA and pKasOp-aur1DEtFCGHA digested with the same restriction enzyme, resulting in pKasOp-aur2ABtFCGHALM and pKasOp-aur1DEtFCGHALM (Fig. 4a).

### Integration of plasmids into *S. coelicolor* M1146 and analysis of rabelomycin production

All integrative plasmids were transformed into *E. coli* ET12567/pUZ8002 strain and subsequently introduced into *S. coelicolor* M1146 (Gomez-Escribano and Bibb 2011) by conjugation (Kieser et al. 2000) with AprR selection. Three independent AprR colonies were picked and sporulated twice on solid SFM medium with Apr and NA and finally on solid SFM medium with Apr. Spores from such plates (areas of about 1 cm^2^ together with the agar medium) were inoculated into 10 ml of CSLS inoculation medium with Apr in 100 ml Erlenmeyer flasks and the seed cultures were grown for 48 h at 270 rpm and 28 ºC. Then, 1 ml of the inoculum was used to inoculate 20 ml of Bennet medium in 100 ml Erlenmeyer flasks and the cultures were grown under the same conditions. After 24, 48, and 72 h, 4 ml culture aliquots were taken, extracted twice with an equal volume of ethyl acetate, dried under a vacuum and finally dissolved in 100 µl methanol. 10 µl aliquots were analysed by HPLC using an OmmiSpher 5 C18 column (5 µm, 250 × 4.6 mm; Varian, Lake Forest, CA, USA) and eluted with a 30-min linear gradient of acetonitrile from 20 to 100% in 0.5% (v/v) acetic acid in water at a flow rate of 1 ml/min. A Shimadzu LC-10A HPLC system with a combined SPD-20A UV–VIS detector (Shimadzu, Kyoto, Japan) with detection at 254 nm and SPD-M14 PDA detector was used. Under these conditions, rabelomycin showed a retention time of 15.3 min and was confirmed by HRESI-MS analysis with a Velos Pro Hybrid Ion Trap-Orbitrap Mass Spectrophotometer (Thermo Scientific, Waltham, MA, USA). Rabelomycin was quantified by comparing the peak area of the corresponding sample at 254 nm with the peak area of a known concentration of purified rabelomycin.

### Bioluminescence measurement

The luciferase reporter plasmid pMU1s* containing the synthetic *luxCDABE* operon (Craney et al. 2007) was used to detect promoter activity of pMU1s-ermEp (Csolleiova et al. 2021) and pMU1s-kasOp (Novakova et al. 2022a, b) in *S. coelicolor* M1146. The plasmids were conjugated into *S. coelicolor* M1146 with AprR selection and eight independent AprR clones from each plasmid were selected for luminescence assay. Spores (approximately 10^5^ CFU) were seeded into individual wells of white 96-well plates (Sigma-Aldrich, Darmstadt, Germany) with 250 µl of solid Bennet medium and incubated at 28 ºC. Luminescence in relative luminescence units (RLU) was determined in a Synergy HT microplate reader (Bio-Tek Instruments, Winooski, VT, USA) every 4 h.

### Disruption of the *S. lavendulae* subsp. *lavendulae* CCM 3239 genes

Mutants with deleted operons *aur1DE* and *aur2AB* in *S. lavendulae* subsp. *lavendulae* CCM 3239 were constructed using the PCR-targeted REDIRECT procedure (Gust et al. 2003). To delete the *aur1DE* genes, the AprR cassette was amplified by PCR using primers Aur1DEdDir and Aur1DEdRev (Additional file 1: Table S2) and plasmid pIJ773 as a template. The resulting PCR-amplified DNA fragment was introduced into cosmid pCosSA5 (Novakova et al. 2010a) in *E. coli* BW25113/pIJ790, resulting in cosmid pCosSA5-aur1DE. Correct replacement of *aur1DE* by the AprR cassette was verified by restriction mapping. To delete the *aur2AB* genes, the AprR cassette was similarly PCR-amplified using primers Aur2ABdDir and Aur2BdRev (Additional file 1: Table S2) and pIJ773 as a template. The resulting PCR-amplified DNA fragment was similarly introduced into pCosSA74 (Novakova et al. 2013), resulting in cosmid pCosSA74-aur3AB. Correct replacement of *aur2AB* by the AprR cassette was verified by restriction mapping. After both recombinant cosmids were passaged through the non-methylating *E. coli* ET12567/pUZ8002 strain, they were introduced into *S. lavendulae* subsp. *lavendulae* CCM 3239 by conjugation. The double crossovers were screened for AprR and Kan sensitivity. Five or four such colonies were identified, resulting in mutant strains *S. lavendulae* Δ*aur1DE::AprR1, 2, 3, 4*, *5* and *S. lavendulae* Δ*aur2AB::AprR1, 2, 3, 4.* To confirm the correct replacement of the genes by the AprR cassettes in chromosome, chromosomal DNA was isolated from the strains and analysed by Southern-blot hybridization (Additional file 1: Figures S4, S5). All clones had similar phenotypes.

### Analysis of auricin production

The production of auricin is described in detail in Novakova et al. (2022b). Briefly, *S. lavendulae* subsp. *lavendulae* CCM 3239, *S. lavendulae* Δ*aur1DE*::AprR, and *S. lavendulae* Δ*aur2AB*::AprR strains were grown in 50 mL of Bennet medium on an orbital shaker at 270 rpm and 28 °C. Auricin was extracted with ethyl acetate from 4 mL culture aliquots at various time points, evaporated to dryness and finally dissolved in 96% ethanol. 10 µl aliquots were analysed by TLC followed by biochromatography using a sensitive *Bacillus subtilis* strain and by HPLC using an OmmiSpher 5 C18 column (5 µm, 250 × 4.6 mm) under the same conditions and detections as described above for analysis of rabelomycin. Under these conditions, auricin showed a retention time of 9 min and was confirmed by HRESI-MS analysis as a molecular ion [M + H]^+^ of *m/z* = 542.2039 with positive mode detection (Kutas et al. 2013).

## Results

### Development of a synthetic biology-based expression system using the initial landomycin biosynthetic genes

The central part of the *aur1* BGC for auricin (Additional file 1: Figure S1) shows synteny with angucycline BGCs and also with incomplete BGC for griseusin (Additional file 1: Figure S2) (Novakova et al. 2002; Matulova et al. 2019). To investigate the biosynthesis of auricin, we aimed to develop a heterologous expression system based on a synthetic biology approach. To test such expression system, we selected the homologous central region of the well-studied landomycin BGC, *lanEFABCDL*, from *S. cyanogenus* S136 (Westrich et al. 1999), encoding the initial PKS II biosynthetic enzymes producing the common angucycline intermediate UWM6, which is converted by LanE to the rabelomycin shunt product (Fig. 1a) (Kharel et al. 2012a; Myronovskyi et al. 2016).

We first tested the activity of two well-known strong promoters, ermEp* (Bibb et al. 1994) and kasOp* (Wang et al. 2013), in a well-known chassis strain for heterologous production of secondary metabolites, *S. coelicolor* M1146, in which four main endogenous BGCs, *act*, *red*, *cda*, *cpk*, were deleted (Gomez-Escribano and Bibb 2011). DNA fragments containing both promoters were previously cloned into the luciferase reporter vector pMU1s*. This integration plasmid, based on the PhiBT1 phage integrase gene and the *attP* site, contains the synthetic operon *luxCDABE* and integrates into a single *attB* site in the *Streptomyces* chromosomes (Craney et al. 2007). Recombinant plasmids pMU1s-ermEp (Csolleiova et al. 2021) and pMU1s-kasOp (Novakova et al. 2022a, b) (Additional file 1: Figure S7a) were conjugated to *S. coelicolor* M1146 and the luminescence of eight independent AprR clones was determined during differentiation on solid Bennet medium. The activity of the kasOp* promoter (on average 304,534 RLU) was approximately threefold higher than the activity of the ermEp* promoter (on average 102,329 RLU) (Additional file 1: Figure S7b). Interestingly, both promoters were significantly more active (approximately 1.6 times) in the *S. coelicolor* M1146 than in the WT *S. coelicolor* M145 strain (Csolleiova et al. 2021; Novakova et al. 2022a, b).

All seven initial landomycin biosynthetic genes (*lanA, lanB, lanC, lanF, lanD, lanL, lanE*) were amplified by PCR to insert the appropriate rare restriction sites and the strong RBS site in front of the respective genes and cloned into a standard cloning vector pBluescript II SK + . The individual genes together with the strong RBS site were then sequentially inserted into the artificial synthetic operon using the cloning strategy shown in Additional file 1: Figure S6. The final artificial operon *lanABCFDLE* was inserted into both pMU1s-ermEp and pMU1s-kasOp expression vectors under the control of ermEp* and kasOp*, replacing the *luxCDABE* operon (Fig. 1b). Both final recombinant vectors, pErmEp-lanABCFDLE and pKasOp-lanABCFDLE, were integrated into the chromosome of *S. coelicolor* M1146 and three independent clones grown in liquid Bennet medium were selected to characterize metabolite production by HPLC at three growth time points. The results revealed one dominant yellow peak, which was missing in the *S. coelicolor* M1146 strain containing the control plasmid pMU1s-kasOp (Fig. 1c). Its Rt value (15.3 min) and spectral properties correspond to rabelomycin (Kharel et al. 2012a; Myronovskyi et al. 2016). In addition, high-resolution ESI MS data confirmed rabelomycin with Mr = 338 (Additional file 1: Figure S8). Rabelomycin production peaked after 3 days of culture and the yield was fivefold higher in the strain containing the construct under the kasOp* promoter (0.91 ± 0.04 mg/L) than in the strain with the construct under the ermEp* promoter (0.18 ± 0.03 mg/L). These results confirmed the activity of both promoters in the luciferase system and suggested to use this “bottom-up” system based on synthetic biology containing the stronger kasOp* promoter for all other studies.

### Initial auricin biosynthetic genes *aur1DEFCGHA* failed to produce any secondary metabolite in the expression system

Based on the high similarity of the initial auricin biosynthetic genes *aur1ACDEFH* with the landomycin genes *lanEFABCDL* (Additional file 1: Figure S2) (Novakova et al. 2002; Matulova et al. 2019), we constructed similar artificial *aur1DEFCGHA* operon using the same strategy as used for the landomycin artificial *lanABCFDLE* operon (Additional file 1: Figure S6). The operon was inserted into pMU1s-kasOp expression vector under the control of kasOp*, replacing the *luxCDABE* operon (Fig. 2a), and the final recombinant plasmid pKasOp-aur1DEFCGHA was integrated into the chromosome of *S. coelicolor* M1146 by conjugation. Three independent clones grown in liquid Bennet medium were selected for characterization of metabolite production by HPLC as described above. However, HPLC analysis of the ethyl acetate extracts revealed no additional peak, compared to the control *S. coelicolor* M1146 strain containing pMU1s-kasOp (Fig. 2b).

To determine which of the auricin genes is responsible for this failure of rabelomycin production, the *aur1DEF* genes (encoding KSα, KSβ, ACP) were replaced by the homologous *lanABC* genes and vice versa. The resulting mixed recombinant plasmids pKasOp-aur1DEFlanFDLE and pKasOp-lanABCaur1CGHA (Fig. 2a) were similarly conjugated into *S. coelicolor* M1146 and the production of secondary metabolites in three independent clones was determined in liquid Bennet medium as described above. All three clones of *S. coelicolor* M1146 containing pKasOp-aur1DEFlanFDLE did not produce rabelomycin. However, all three clones of *S. coelicolor* M1146 containing pKasOp-lanABCaur1CGHA produced yellow rabelomycin (as confirmed by spectrum and high-resolution ESI MS analysis as described above) at a similar level (1.24 ± 0.25 mg/L) as original pKasOp-lanABCFDLE (Fig. 2b). These results indicated that some of the initial *aur1DEF* genes are not active.

### Distant ***aur2AB*** genes encoding KSα and KSβ are essential for auricin biosynthesis, but not ***aur1DE***

**Fig. 5:**
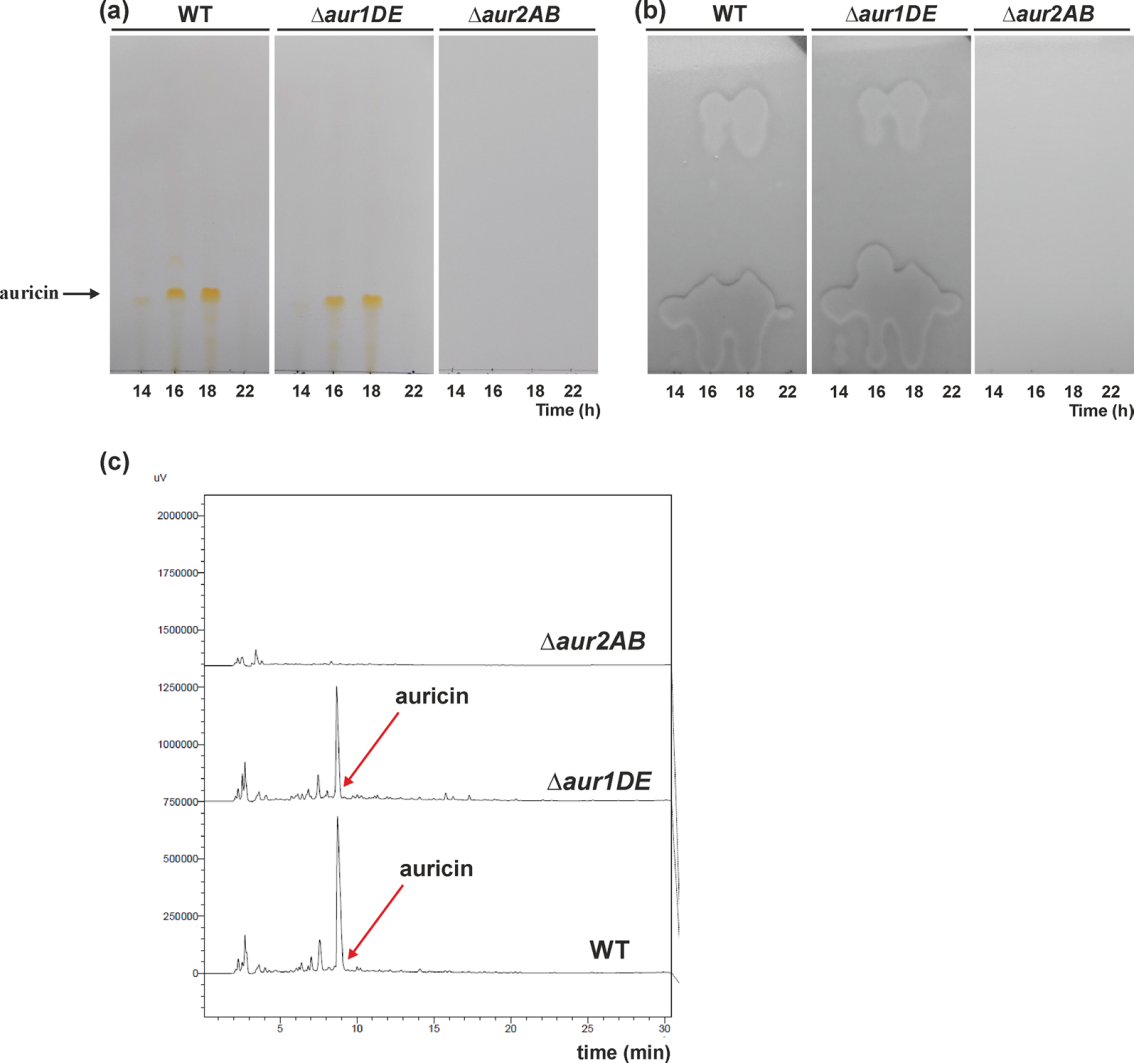
Detection of auricin in 10 µl ethyl acetate extracts from *S. lavendulae* subsp. *lavendulae* CCM 3239 (WT), *aur1DE* mutant (Δ*aur1DE*), and *aur2AB* mutant (Δ*aur2AB*) strains grown in Bennet medium to the indicated time points by TLC **a** followed by biochromatography with *B. subtilis* test strain **b** as is described in Material and Methods. **c** HPLC analysis of 10 µl ethyl acetate extracts from the indicated strain grown for 16 h in Bennet medium (details described in Material and Methods). The arrow indicates the position of the auricin peak

To examine the role of both *aur1DE* genes, encoding KSα and KSβ, in auricin biosynthesis, their coding region was deleted in *S. lavendulae* subsp. *lavendulae* CCM 3239 using the REDIRECT PCR-targeting system and replaced with the AprR cassette (Gust et al. 2003). Correct disruption was verified by Southern blot hybridization analysis (Additional file 1: Figure S4). Auricin production of this *S. lavendulae* Δ*aur1DE* mutant was investigated as described previously (Kutas et al. 2013). Interestingly, deletion of the *aur1DE* genes had no effect on auricin production. The inhibition zones corresponding to auricin in the mutant were similar to wild-type *S. lavendulae* subsp. *lavendulae* CCM 3239 strain for all investigated time points (Fig. 5a, b). In addition, the level of auricin determined by HPLC was similar in both wild-type and mutant strains (Fig. 5c). This was surprising because deletion of the entire *aur1* BGC from *sa22* to *aur1V* (Additional file 1: Figure S1) resulted in the absence of auricin (Novakova et al. 2013).

The partial type II PKS BGC *aur2*, which contains two translationally-coupled genes *aur2A* and *aur2B* encoding KSα and KSβ, is located in the distal region of the *aur1* BGC core. However, this BGC lacks the gene encoding ACP (Additional file 1: Figure S1). Both *aur2AB* genes were similarly deleted from *S. lavendulae* subsp. *lavendulae* CCM 3239 and the correct integration was verified by Southern blot hybridization analysis (Additional file 1: Figure S5). Auricin production in the *S. lavendulae* Δ*aur2AB* mutant was examined as described above. Contrary to previous results, deletion of the *aur2AB* genes resulted in the absence of auricin (Fig. 5a, b, c). Therefore, these results surprisingly indicated that these *aur2AB* genes, located in the distal region of the *aur1* BGC, are essential for auricin biosynthesis, rather than the *aur1DE* genes located in the core *aur1* BGC.

Phylogenetic analysis of both Aur1DE and Aur2AB with several representative KSα and KSβ proteins from the major groups of aromatic polyketides revealed that both Aur1D and Aur1E are in the angucycline clade, as we found previously (Matulova et al. 2019), but both Aur2A and Aur2B were clearly placed in the aureolic acid clade (Additional file 1: Figure S9). The most similar were MtmP (KSα) and MtmK (KSβ) from mithramycin BGC (Additional file 1: Figures S10, S11). Mithramycin, auricin, and landomycin all have a decaketide aglycone. Therefore, Aur2AB may be suitable candidates for the initial biosynthesis of auricin.

### The *aur2AB* genes are active in the expression system together with the *aur1F* gene encoding ACP, which needs activation by the PPTase encoded by *aur1L*

To investigate the functionality of both *aur2AB* genes in the previous expression system, they were amplified as an operon and used to replace the homologous *lanAB* genes in pKasOp-lanABCFDLE, resulting in the recombinant plasmid pKasOp-aur2ABtlanCFDLE. Similarly, both *aur1DE* genes were amplified as an operon and used to replace the *lanAB* genes, resulting in the recombinant plasmid pKasOp-aur1DEtlanCFDLE (Fig. 3a). Both plasmids were conjugated into *S. coelicolor* M1146 and three independent clones grown in liquid Bennet medium were selected for characterization of metabolite production by HPLC as described above. All three *S. coelicolor* M1146 clones containing pKasOp-aur1DEtlanCFDLE did not produce rabelomycin (similar to the previous pKasOp-aur1DEFlanFDLE construct as described above). However, all three *S. coelicolor* M1146 clones containing pKasOp-aur2ABtlanCFDLE produced yellow rabelomycin (as confirmed by spectrum and high-resolution ESI MS analysis as described above) at a significantly higher level (2.71 ± 0.32 mg/L) than original pKasOp-lanABCFDLE (Fig. 3b). These results indicated that both *aur2AB* genes are active in the heterologous expression system, in contrast to the *aur1DE* genes.

To examine both *aur2AB* genes with the homologous auricin biosynthetic gene, they were used to replace the *aur1DE* genes in pKasOp-aur1DEFCGHA, resulting in the recombinant plasmid pKasOp-aur2ABtFCGHA. Similarly, as a control, both *aur1DE* genes (as original operon) were used to replace the separated *aur1DE* genes in pKasOp-aur1DEFCGHA, resulting in the recombinant plasmid pKasOp-aur1DEtFCGHA (Fig. 3a). Both plasmids were conjugated into *S. coelicolor* M1146 and three independent clones grown in liquid Bennet medium were selected for characterization of metabolite production by HPLC as described above. In the case of pKasOp-aur1DEtFCGHA, there was no production of any additional peak, compared to the control *S. coelicolor* M1146 strain containing pMU1s-ermEp4, as expected. All three *S. coelicolor* M1146 clones containing pKasOp-aur2ABtFCGHA produced yellow rabelomycin (as confirmed by spectrum and high-resolution ESI MS analysis as described above), but at a lower level (0.4 ± 0.05 mg/L) than original pKasOp-lanABCFDLE (Fig. 3b).

The *aur2* BGC does not contain a gene encoding ACP; therefore the *aur1F* gene should be involved in auricin biosynthesis. The *aur1* BGC contains the *aur1L* gene encoding PPTase (Additional file 1: Figure S1). This protein is essential for ACP activation. The genome of the host strains *S. coelicolor* M1146 contains three genes encoding PPTases (Lu et al. 2008). It is possible that *aur1F*, unlike the homologous *lanC*, is weakly activated by some heterologous PPTase(s) from the host strain *S. coelicolor* M1146. Therefore, we inserted the *aur1L* gene together with the downstream *aur1M* gene encoding MCAT, which is essential for charging ACP with an extender malonyl unit, into pKasOp-aur2ABtFCGHA, resulting in pKasOp-aur2ABtFCGHALM (Fig. 4a). All three *S. coelicolor* M1146 clones containing this plasmid produced a significantly higher level of rabelomycin, on average about 31-fold (12.4 ± 1.07 mg/L), compared to the construct without the *aur1LM* genes (Fig. 4b). However, similar insertion of both *aur1LM* genes into pKasOp-aur1DEtFCGHA, which resulted in pKasOp-aur1DEtFCGHALM (Fig. 4a), resulted in only traces of rabelomycin production (Fig. 4b). These results indicated that the ACP encoded by the *aur1F* gene is specifically activated by the PPTase encoded by the *aur1L* gene, located in the *aur1* BGC.

## Discussion

Synthetic biology-based approaches have been successfully used to produce native or modified natural products in various hosts (Ajikumar et al. 2010; Ro et al. 2006). These tools have also recently been used in type II PKS biosynthetic strategies to produce various aromatic polyketides in *Streptomyces* spp. (Wang et al. 2022; Zhu et al. 2021). In this report, we constructed a plasmid system based on combinatorial synthetic biology to study the initial steps of auricin biosynthesis. The central part of the *aur1* BGC is located in the *aur1ABCDEFHHIJKLMN* operon (Additional file 1: Figure S1), which is controlled by the first biosynthetic promoter *aur1Ap* (Kormanec et al. 2014; Novakova et al. 2011). Although this central part is homologous to several angucycline antibiotics (Additional file 1: Figure S2), the structure of the auricin aglycone is similar to spiroketal pyranonaphthoquinone griseusins (Additional file 1: Figure S3) (Matulova et al. 2019). Although several griseusins from various strains have already been characterized, their biosynthesis is still unknown. Therefore, we decided to study the biosynthesis of auricin using a synthetic biology approach. Unlike previously published type II PKS synthetic biology systems (Wang et al. 2022; Zhu et al. 2021), our established system was based on single gene insertions under the control of strong RBS (Craney et al. 2007) in a synthetic operon using unique restriction sites, absent in all genes examined (Additional file 1: Figure S3), which can allow simple replacement of any gene in the operon with some other homologous gene. To test this system, we first selected a homologous portion of the landomycin BGC (*lanEFABCDL*) from *S. cyanogenus* S136 (Westrich et al. 1999) (Additional file 1: Figure S2), encoding the initial PKS II biosynthetic enzymes producing the shunt product rabelomycin (Kharel et al. 2012a; Myronovskyi et al. 2016). The final artificial operon *lanABCFDLE* was inserted into the phage PhiBT1-based integration vector under the control of the ermEp* and kasOp* promoters and integrated into the chromosome of the host strain *S. coelicolor* M1146. Rabelomycin was correctly produced, but its level was fivefold higher with the kasOp* promoter than with the ermEp* promoter (Fig. 1). It was consistent with our promoter assays using a luciferase reporter system (Additional file 1: Figure S7). Therefore, the results validated the system. However, when homologous auricin biosynthetic genes *aur1DEFCGHA* were similarly inserted using this system under the kasOp* promoter in *S. coelicolor* M1146, no rabelomycin or other secondary metabolite was produced. Several other gene exchange experiments in this system confirmed that this failure was caused by inactive *aur1DE* genes encoding KSα and KSβ. Comparison of Aur1D and Aur1E with the corresponding KSα and KSβ subunits from other angucycline BGCs showed several different residues (30 in Aur1D and 15 in Aur1E), which were conserved in all other KSs (Additional file 1: Figures S12, S13). Although the Cys-His-His catalytic triad (Keatinge-Clay et al. 2004) was present in Aur1D, these different residues may explain the inactivity of these proteins. Interestingly, two other homologous *aur2AB* genes in the adjacent region to the *aur1* BGC were active in the system to produce expected rabelomycin together with *lanCFDLE* or *aur1FCGHA*. Moreover, deletion of *aur1DE* and *aur2AB* in *S. lavendulae* subsp. *lavendulae* CCM 3239 clearly confirmed that only the *aur2AB* genes are essential for auricin biosynthesis. However, rabelomycin production was significantly lower with the *aur2AB* and *aur1FCGHA* genes than with the homologous *lanCFDLE* genes. Insertion of the PPTase-encoding *aur1L* together with the MCAT–encoding *aur1M* gene of the *aur1* BGC into this construct significantly induced rabelomycin production, indicating that the Aur1L PPTase is essential for Aur1F ACP activation.

These results were surprising. To our knowledge, this is the first example that two BGCs can interact in the biosynthesis of a secondary metabolite. Aur2A KSα and Aur2B KSβ from the *aur2* BGC, together with Aur1F ACP from the *aur1* BGC, appear to be essential for the biosynthesis of the initial auricin decaketide, which can be further modified by AurG KR, Aur1H ARO, Aur1A OXY, and Aur1C CYC, at least in the artificial synthetic biology system in *S. coelicolor* M1146. Whether it occurs in *S. lavendulae* subsp. *lavendulae* CCM 3239 should be further investigated. Regarding the interesting griseusin-like structure (Matulova et al. 2019), we hypothesize that only a few genes from *aur1* and *aur2* BGCs (Additional file 1: Figure S1) will be required for the complete biosynthesis of the auricin aglycone. It could be similar as to the biosynthesis of the sugar moiety of auricin, D-forosamine (Bekeova et al. 2016). Its biosynthesis similarly depends on gene products from the central *aur1* BGC (*aur1TQSV*) and several genes outside this region (*sa50, sa52, sa59, sa46, sa53*) but near the *aur2* BGC. Therefore, this new optimized synthetic biology system will be further used to solve this interesting biosynthesis of auricin aglycone. These experiments are ongoing in our laboratory.

In conclusion, we have constructed a synthetic biology-based plasmid system utilizing the strong kasOp* promoter, RBS, and phage PhiBT1-based integration vector that can be used to investigate the biosynthesis of aromatic polyketides. The system was validated with initial biosynthetic genes *lanABCFDLE* from the landomycin BGC, leading to the production of rabelomycin after its integration into the host strain *S. coelicolor* M1146. However, the *aur1DEFCGHA* homologous genes from the auricin *aur1* BGC were not active in the system for rabelomycin production. This failure was caused by inactive *aur1DE* genes encoding KSα and KSβ. Their replacement with homologous *aur2AB* genes from the adjacent *aur2* BGC resulted in production of rabelomycin. In addition, insertion of two additional genes from the *aur1* BGC, *aur1L* encoding PPTase and *aur1M* encoding MCAT, substantially induced rabelomycin production, indicating that Aur1L PPTase is essential for Aur1F ACP activation. These results suggest an interesting communication of two adjacent BGCs, *aur1* and *aur2*, in the biosynthesis of the initial structure of auricin aglycone.

### Supplementary Information

**Additional file 1: Table S1.** Bacterial strains and plasmids used in this study. **Table S2.** Oligonucleotides used in this study. Cloning sites are underlined. **Figure S1.** Genetic organization of the *aur1* BGC for auricin and its flanking regions. Arrows indicates the position and direction of expression of indivilual genes. Details of the genes and their products are desribed in Genbank Acc. No. KJ396772. **Figure S2.** Comparison of auricin *aur1* BGC core gene organization from *S. lavendulae* subsp. *lavendulae* CCM 3239 (Kormanec et al. 2014) with incomplete griseusin BGC from *S. griseus* K-63 (Yu et al. 1994) and other angucycline BGCs*.* Homologous *g*enes are indicated by arrows with the same colour. BGC accession numbers are: *Streptomyces* sp. PGA64 gaudimycin (*pga*) BGC (AY034378), *S. ambofaciens* ATCC 23877 kinamycin (*alp*) BGC (AY338477), *S. cyanogenus* S136 landomycin (*lan*) BGC (AF080235), *S. fradiae* Tu2717 urdamycin (*urd*) BGC (X87093), *S. antibioticus* ATCC 11891 oviedomycin (*ovm*) BGC (AJ632203), *S. antibioticus* Tu6040 simocyclinone (*sim*) BGC (AF324838), *S. griseoflavus* Goe 3592 gilvocarcin (*gil*) BGC (AY233211), *Streptomyces* sp. SCC-2136 angucyclines Sch 47554 and Sch 47555 (*sch*) BGC (AJ628018). The conserved central regions encode oxygenase (OXY), angucycline-specific cyclase (CYC), ketosynthase α (KSα), ketosynthase β (KS β), acyl carrier protein (ACP), ketoreductase (KR), and aromatase (ARO). In three BGCs, this region is interrupted by a gene (*aur1B*, *pgaY*, *alpJ*) encoding a conserved TetR family regulator. **Figure S3.** Structure of auricin, 3´-O-α-D-forosaminyl-( +)-griseusin A, 4´-dehydro-deacetylgriseusin A (griseusin C), and landomycin E (angucycline group), and medermycin (pyranonaphthoquinone group). Numbering of positions is according to the published data for each compound. **Figure S4. a** Genetic organization of the *aur1* BGC around the *aur1DE* genes in wild-type *S. lavendulae* subsp. *lavendulae* CCM 3239 (Kormanec et al. 2014; Matulova et al. 2019) and the disrupted strain *S. lavendulae* Δ*aur1DE::AprR.* Coloured arrows indicate individual genes; green correspond to the *aur1* BGC and red to the regulatory gene. The yellow box with the blue arrow indicates the AprR *aac3(IV)* gene with the *oriT* origin of transfer (red column) from pIJ773 (Gust et al. 2003). The blue bar below the maps indicates the position of the probe 1 used for Southern hybridization analysis. Relevant restriction sites are included. (**b**) Southern blot hybridization analysis of five *S. lavendulae* Δ*aur1DE::AprR* clones and wild-type *S. lavendulae* subsp. *lavendulae* CCM 3239 (WT) as a control. 1 µg of DNA from the respective strain was digested with the indicated restriction endonucleases and separated by electrophoresis in a 0.8% (w/v) agarose gel. After transfer to a Hybond N membrane, hybridization was performed according to the standard DIG protocol as desribed in materials and methods using DIG-labelled probe 1 covering the *aur1B* gene. *Bst*EII-digested lambda DNA was used as a size standard. **Figure S5. a** Genetic organization of the type II PKS BGC *aur2* around two translationally-coupled genes *aur2A* and *aur2B* encoding KSα and KSβ, in wild-type *S. lavendulae* subsp. *lavendulae* CCM 3239 (Kormanec et al. 2014; Matulova et al. 2019) and the disrupted strain *S. lavendulae* Δ*aur2AB::AprR.* Coloured arrows indicate individual genes; purple correspond to the *aur2* BGC and yellow to the pSA3239 replication gene. The yellow box with the blue arrow indicates the AprR *aac3(IV)* gene with the *oriT* origin of transfer (red column) from pIJ773 (Gust et al. 2003). The blue bar below the maps indicates the position of the probe 2 used for Southern hybridization analysis. Relevant restriction sites are included. (**b**) Southern blot hybridization analysis of four *S. lavendulae* Δ*aur2AB::AprR* clones and wild-type *S. lavendulae* subsp. *lavendulae* CCM 3239 (WT) as a control. 1 µg of DNA from the respective strain was digested with the indicated restriction endonucleases and separated by electrophoresis in a 0.8% (w/v) agarose gel. After transfer to a Hybond N membrane, hybridization was performed according to the standard DIG protocol as desribed in materials and methods using DIG-labelled probe 2 covering the *aur2BCD* gene region. *Bst*EII-digested lambda DNA was used as a size standard. **Figure S6.** Cloning strategy to create the artificial operon *lanABCFDLE*. Each individual *lan* gene was PCR amplified together with a strong RBS site and cloned into pBluescript II SK to obtain plasmids pBS-lanA, pBS-lanB, pBS-lanC, pBS-lanF, pBS-lanD, pBS-lanL, and pBS-lanE. Subsequently, the genes were inserted in the final operon according to the scheme to obtain the final plasmid pBS-lanABCFDLE. Sequences upstream of individual genes are shown with RBS from pMU1s* (Craney et al. 2007) in green and the ATG codon underlined. The relevant restriction sites are indicated. **Figure S7. a** Schemes of the *luxCDABE* reporter PhiBT1 phage integration plasmid pMU1s* (Craney et al. 2007) and recombinant plasmids containing the ermEp* or kasOp* promoter. Arrows indicate the position and orientation of individual genes, and bent arrows indicate the position of promoters. The plasmid backbone contains the AprR *aac3(IV)* gene (blue arrow), the *oriT* origin of transfer (red bar), the *phiBT1int* integrase gene (green arrow) together with the *attP* site (green bar), the *fdT* terminator (dark grey box), and the *E. coli* origin of replication *ColE1* (light grey box) from plasmid pMU1s* (Craney et al. 2007). Only relevant restriction nuclease sites are shown. **b** The luciferase activity of the *luxCDABE* operon after fusion with the corresponding promoters. The plasmids were introduced into *S. coelicolor* M1146 by conjugation and the luminescence of eight clones from each construct was determined in relative luminescence units (RLU) after growth and differentiation on solid Bennet medium in 96-well plates at the indicated time points. Each time point represents the mean, and the error bar indicates the standard deviation from the mean. Black arrows above the graph indicate developmental stages. **Figure S8.** High resolution ESI MS spectrum in positive mode for the rabelomycin peak (*m/z* = 339.08641 [M = H] + , calculated mass is 339.08631). **Figure S9.** Phylogenetic trees of selected ketosynthase α (KSα) and ketosynthase β (KSβ), which represent basic groups of aromatic polyketides. The Neighbour Joining method (Saiton and Nei 1987) was used to construct these trees based on the comparison of KSα (Aur1D, Aur2A) and KSβ (Aur1E, Aur2B) *from S. lavendulae* subsp. *lavendulae* CCM 3239 with the selected KSα and KSβ from the basic groups of aromatic polyketides. Protein alignments and their desriptions are shown in supplementary figures S8 and S9 below. **Figure S10.** Comparison of the amino acid sequence of KSα (Aur1D, Aur2A) *from S. lavendulae* subsp. *lavendulae* CCM 3239 with selected KSα proteins from the basic groups of aromatic polyketides. Protein sequences (and corresponding accession numbers) are as follows: auricin Aur2A (AIE41912), mithramycin MtmP (CAA61989), granaticin Gra-ORF1 (CAA09653), qinimycin Qin-ORF19 (WP_058047374), medermycin Med-ORF1 (BAC79044), actinorhodin ActI-ORF1 (CAC44200), alnumycin AlnL (ACI88861), frenolicin FrnL (AAC18107), kinamycin AlpA (AAR30152), jadomycin JadA (AAB36562), landomycin LanA (AAD13536), gaudimycin PgaA (AAK57525), simocyclinone SimA1 (AAK06784), urdamycin UrdA (CAA60569), auricin Aur1D (AAX57191), griseusin Gris-ORF1 (CAA54860), elloramycin ElmK (CAP12600), tetracenomycin TcmK (AAA67515), pradimicin PdmA (ABM21747), daunorubicin Dau-ORFA (AAA87618), doxorubicin DpsA (AAA65206), oxytetracycline OxyA (AAZ78325). **Figure S11.** Comparison of the amino acid sequence of KSβ (Aur1E, Aur2B) *from S. lavendulae* subsp. *lavendulae* CCM 3239 with selected KSβ proteins from the basic groups of aromatic polyketides. Protein sequences (and corresponding accession numbers) are as follows: elloramycin ElmL (CAP12601), tetracenomycin TcmL (AAA67516), pradimicin PdmB (ABM21748), jadomycin JadB (AAB36563), urdamycin UrdB (CAA60570), kinamycin AlpB (AAR30151), auricin Aur1E (AAX57192), griseusin Gris-ORF2 (CAA54859), landomycin LanB (AAD13537), gaudimycin PgaB (AAK57526), simocyclinone SimA2 (AAK06785), daunorubicin Dau-ORFB (AAA87619), doxorubicin DpsB (AAA65207), actinorhodin ActI-ORF2 (CAC44201), qinimycin Qin-ORF18 (WP_058047373), granaticin Gra-ORF2 (CAA09654), medermycin Med-ORF2 (BAC79045), alnumycin AlnM (ACI88862), frenolicin FrnM (AAC18108), auricin Aur2B (AIE41911), mithramycin MtmK (CAA61990), oxytetracycline OxyB (AAZ78326). **Figure S12.** Comparison of the amino acid sequence of Aur1D *from S. lavendulae* subsp. *lavendulae* CCM 3239 with the corresponding KSα proteins from the angucycline BGCs. Protein sequences (and corresponding accession numbers) are as follows: PgaA (AAK57525), LanA (AAD13536), AlpA (CAJ87874), JadA (AAB36562), UrdA (caa60569), SimA1 (AAK06784, Aur1D (AAX57191). Amino acid residues highlighted in green correspond to the Cys-His-His catalytic triad (Keatinge-Clay et al. 2004), highlighted in yellow different residues in Aur1D that were conserved in all other KSs. **Figure S13.** Comparison of the amino acid sequence of Aur1E *from S. lavendulae* subsp. *lavendulae* CCM 3239 with the corresponding KSβ proteins from the angucycline BGCs. Protein sequences (and corresponding accession numbers) are as follows: AlpB (CAJ87873), JadB (AAB36563), UrdB (CAA60570), SimA2 (AAK06785), PgaB (AAK57526), Aur1E (AAX57192), LanB (AAD13537). Amino acid residues highlighted in green correspond to the altered catalytic triad in CLF (Keatinge-Clay et al. 2004), highlighted in yellow different residues in Aur1E that were conserved in all other KSs.

## Data Availability

The authors can confirm that all relevant data are included in the article and/or its supplementary information files.

## Abbreviations

ACP: Acyl carrier protein
Amp: Ampicillin
Apr: Apramycin
AprR: pr resistance
ARO: Aromatase
Clm: Chloramphenicol
CSLS: Corn steep liquor-sucrose
CYC: Cyclase
BGC: Biosynthetic gene cluster
DIG: Digoxigenin
Kan: Kanamycin
Kb: Kilobase
KR: Ketoreductase
KSα: Ketosynthases α
KSβ: Ketosynthases β
LB: Luria–Bertani
MCAT: Malonyl-CoA:ACP transacylase
NA: Nalidixic acid
OXY: Oxygenase
PKS: Polyketide synthase
PPTase: 4-phosphopantetheinyl transferase
RBS: Ribosome binding site
RLU: Relative luminescence unit
WT: Wild type
