# Supplementary material for: Investigating the initial steps of auricin biosynthesis using synthetic biology
Source: AMB Express. 2023 Aug 8;13:83. doi: 10.1186/s13568-023-01591-2 (PMC10409956; doi:10.1186/s13568-023-01591-2)
Supplement: Supplementary file 1 — Additional file 1: Table S1. Bacterial strains and plasmids used in this study. Table S2. Oligonucleotides used in this study. Cloning sites are underlined. Figure S1. Genetic organization of the aur1 BGC for auricin and its flanking regions. Arrows indicates the position and direction of expression of indivilual genes. Details of the genes and their products are desribed in Genbank Acc. No. KJ396772. Figure S2. Comparison of auricin aur1 BGC core gene organization from S. lavendulae subsp. lavendulae CCM 3239 (Kormanec et al. 2014) with incomplete griseusin BGC from S. griseus K-63 (Yu et al. 1994) and other angucycline BGCs. Homologous genes are indicated by arrows with the same colour. BGC accession numbers are: Streptomyces sp. PGA64 gaudimycin (pga) BGC (AY034378), S. ambofaciens ATCC 23877 kinamycin (alp) BGC (AY338477), S. cyanogenus S136 landomycin (lan) BGC (AF080235), S. fradiae Tu2717 urdamycin (urd) BGC (X87093), S. antibioticus ATCC 11891 oviedomycin (ovm) BGC (AJ632203), S. antibioticus Tu6040 simocyclinone (sim) BGC (AF324838), S. griseoflavus Goe 3592 gilvocarcin (gil) BGC (AY233211), Streptomyces sp. SCC-2136 angucyclines Sch 47554 and Sch 47555 (sch) BGC (AJ628018). The conserved central regions encode oxygenase (OXY), angucycline-specific cyclase (CYC), ketosynthase α (KSα), ketosynthase β (KS β), acyl carrier protein (ACP), ketoreductase (KR), and aromatase (ARO). In three BGCs, this region is interrupted by a gene (aur1B, pgaY, alpJ) encoding a conserved TetR family regulator. Figure S3. Structure of auricin, 3´-O-α-D-forosaminyl-( +)-griseusin A, 4´-dehydro-deacetylgriseusin A (griseusin C), and landomycin E (angucycline group), and medermycin (pyranonaphthoquinone group). Numbering of positions is according to the published data for each compound. Figure S4. a Genetic organization of the aur1 BGC around the aur1DE genes in wild-type S. lavendulae subsp. lavendulae CCM 3239 (Kormanec et al. 2014; Matulova et al. 2019) and the disrupted strain S [file 13568_2023_1591_MOESM1_ESM.pdf]

AMB Express

Supporting information for:

Investigating the initial steps of auricin biosynthesis using synthetic biology

Dominika Csolleiova[1], Rachel Javorova[1], Renata Novakova[1], Lubomira Feckova[1], Maria Matulova[2], Filip Opaterny[1], Bronislava Rezuchova[1], Beatrica Sevcikova[1], Jan Kormanec[1*]

[1] Institute of Molecular Biology, Slovak Academy of Sciences, 845 51 Bratislava, Slovak Republic.
[2] Institute of Chemistry, Slovak Academy of Sciences, 845 38 Bratislava, Slovak Republic.

[*] *Corresponding author:* Jan Kormanec, Institute of Molecular Biology, v.v.i., Slovak Academy of Sciences, Dubravska cesta 21, 845 51 Bratislava, Slovak Republic.
Tel: +421 2 59307419, Fax: +421 2 59307416, E-mail: jan.kormanec@savba.sk
ORCID ID: 0000-0003-0377-6413

Table S1. Bacterial strains and plasmids used in this study

Strain or plasmid	Genotypes and relevant characteristics[a]	Reference or source
Strains		
S. lavendulae subsp. *lavendulae* CCM 3239	Wild-type	(Busche et al. 2018)
S. coelicolor M1146	Δ*act* Δ*red* Δ*cda* Δ*cpk*	(Gomez-Escribano and Bibb 2011)
S. lavendulae Δ*aur1DE::AprR*	Apr^{R}, the 2.48-kb *aur1DE* region replaced by AprR in pSA3239	this study
S. lavendulae Δ*aur2AB::AprR*	Apr^{R}, the 2.53-kb *aur2AB* region replaced by AprR in pSA3239	this study
E. coli DH5α	F^{-} *supE44* Δ*lacU169* (ϕ80d*lacZ*ΔM15) *hsdR17 recA1 endA1 gyrA96 thi-1 relA1*, host strain for plasmid cloning and propagation	Invitrogen
E. coli ET12567/pUZ8002	non-methylating strain used for conjugation from *E. coli* to *Streptomyces*, *recF dam dcm hsdC*, Clm^{R} Str^{R} Tet^{R} Kan^{R}	(Kieser et al. 2000)
E. coli BW25113	Δ(*araD-rhaB*) Δ*lacZ4787* (*::rrnB-4*) *lacIp-40000(*lacI^{Q}*)* λ^{-} *rpoS369*(Am) *rph-1* Δ(*rhaD-rhaB*)568 *hsdR514*, host strain for λ RED recombination	(Gust et al. 2003)
Plasmids		
H2-26	Apr^{R}, *ori* colE1, *E. coli* cosmid vector pOJ446 containing landomycin BGC of *S. cyanogenus* S136	(Westrich et al. 1999)
pCosSA5	Kan^{R}, *ori* colE1, the 41-kb *Sau*3AI DNA fragment from pSA3239 in sCos-1	(Novakova et al. 2010)
pCosSA5-aur1DE	Kan^{R}, Apr^{R}, *ori* colE1, the 2.48-kb *aur1DE* region replaced by AprR in pCosSA5	this study
pCosSA74	Kan^{R}, *ori* colE1, the 36-kb *Sau*3AI DNA fragment from pSA3239 in sCos-1	(Novakova et al. 2013)
pCosSA74-aur2AB	Kan^{R}, Apr^{R}, *ori* colE1, the 2.53-kb *aur2AB* region replaced by AprR in pCosSA74	this study
pBluescript II SK	Amp^{R}, *ori* colE1, *E. coli* plasmid cloning vector	Stratagene
pBS-lanA	Amp^{R}, *ori* colE1, the *lanA* gene in pBluescript II SK	this study
pBS-lanB	Amp^{R}, *ori* colE1, the *lanB* gene in pBluescript II SK	this study
pBS-lanC	Amp^{R}, *ori* colE1, the *lanC* gene in pBluescript II SK	this study
pBS-lanF	Amp^{R}, *ori* colE1, the *lanF* gene in pBluescript II SK	this study
pBS-lanD	Amp^{R}, *ori* colE1, the *lanD* gene in pBluescript II SK	this study
pBS-lanL	Amp^{R}, *ori* colE1, the *lanL* gene in pBluescript II SK	this study
pBS-lanE	Amp^{R}, *ori* colE1, the *lanE* gene in pBluescript II SK	this study
pBS-aur1D	Amp^{R}, *ori* colE1, the *aur1D* gene in pBluescript II SK	this study
pBS-aur1E	Amp^{R}, *ori* colE1, the *aur1E* gene in pBluescript II SK	this study

pBS-aur1F	Amp^R, *ori* colE1, the *aur1F* gene in pBluescript II SK	this study
pBS-aur1C	Amp^R, *ori* colE1, the *aur1C* gene in pBluescript II SK	this study
pBS-aur1G	Amp^R, *ori* colE1, the *aur1G* gene in pBluescript II SK	this study
pBS-aur1H	Amp^R, *ori* colE1, the *aur1H* gene in pBluescript II SK	this study
pBS-aur1A	Amp^R, *ori* colE1, the *aur1A* gene in pBluescript II SK	this study
pBS-aur2ABt	Amp^R, *ori* colE1, the *aur2AB* operon in pBluescript II SK	this study
pBS-aur1DEt	Amp^R, *ori* colE1, the *aur1DE* operon in pBluescript II SK	this study
pBS-aur1LMt	Amp^R, *ori* colE1, the *aur1LM* operon in pBluescript II SK	this study
pIJ773	Amp^R, Apr^R, *ori* colE1, *E. coli* plasmid vector	(Gust et al. 2003)
pMU1s*	Apr^R, PhiBT1-based *Streptomyces* integration vector	(Craney et al. 2007)
pMU1s-ermEp4	Apr^R, PhiBT1-based *Streptomyces* integration vector with *ermEp** promoter	(Csolleiova et al. 2021)
pMU1s-kasOp1	Apr^R, PhiBT1-based *Streptomyces* integration vector with *kasOp** promoter	(Novakova et al. 2022a)
pErmEp-lanABCFDLE	Apr^R, synthetic *lanABCFDLE* operon cloned under the *ermEp** promoter in pMU1s-ermEp4	this study
pKasOp-lanABCFDLE	Apr^R, synthetic *lanABCFDLE* operon cloned under the *kasOp** promoter in pMU1s-kasOp	this study
pKasOp-aur1DEFCGHA	Apr^R, synthetic *aur1DEFCGHA* operon cloned under the *kasOp** promoter in pMU1s-kasOp	this study
pKasOp-aur1DEFlanFDLE	Apr^R, synthetic mixed *aur1DEFlanFDLE* operon cloned under the *kasOp** promoter in pMU1s-kasOp	this study
pKasOp-lanABCaur1CGHA	Apr^R, synthetic mixed *lanABCaur1CGHA* operon cloned under the *kasOp** promoter in pMU1s-kasOp	this study
pKasOp-aur2ABtFCGHA	Apr^R, synthetic mixed *aur2ABtaur1FCGHA* operon cloned under the *kasOp** promoter in pMU1s-kasOp	this study
pKasOp-aur2ABtlanCFDLE	Apr^R, synthetic mixed *aur2ABtlanCFDLE* operon cloned under the *kasOp** promoter in pMU1s-kasOp	this study
pKasOp-aur1DEtFCGHA	Apr^R, synthetic mixed *aur1DEtFCGHA* operon cloned under the *kasOp** promoter in pMU1s-kasOp	this study
pKasOp-aur1DEtlanCFDLE	Apr^R, synthetic mixed *aur1DEtlanCFDLE* operon cloned under the *kasOp** promoter in pMU1s-kasOp	this study
pKasOp-aur2ABtFCGHALM	Apr^R, synthetic mixed *aur2ABtaur1FCGHALM* operon cloned under the *kasOp** promoter in pMU1s-kasOp	this study
pKasOp-aur1DEtFCGHALM	Apr^R, synthetic mixed *aur1DEtFCGHALM* operon cloned under the *kasOp** promoter in pMU1s-kasOp	this study

[a] Clm^{R}, chloramphenicol resistance; Amp^{R}, ampicillin resistance; Apr^{R}, apramycin resistance; Kan^{R}, kanamycin resistance; Str^{R}, streptomycin resistance; Tet^{R}, tetracycline resistance

Table S2. Oligonucleotides used in this study. Cloning sites are underlined.

Oligonucleotide	Sequence (5′ → 3′)
Aur1Bdir	CCCGTACGAGCACCTCAAGAGC
Aur1Brev	GAAGTCCGCGGGTGGTGTCAGG
Aur2Cdir	GACCTCTTGCAGCTGGGCGACG
Aur2Crev	GCCGAGATCTACGGAACCGTCG
Aur1DEdDir	GCGGTGCGGACCGAGGCCGTGCGCGGCCGTACGGCCACCATTCCGGGGATCCGTCGACC
Aur1DEdRev	CGAGGGGAGGGCCGTACCGGTGACCCGTCGGGTAGTGATTGTAGGCTGGAGCTGCTTC
Aur2ABdDir	CGACAGCACAGCAGAAGGACTCACCGTGAAGGACACCCAATTCCGGGGATCCGTCGACC
Aur2BdRev	GTTAATGCGCGGCGCGGACCACCATGGCCGCGTTGAAGCTGTAGGCTGGAGCTGCTTC
-47	CGCCAGGGTTTTCCCAGTCACGAC
-48	AGCGGATAACAATTTCACACAGGA
LanAdir	CCCCCTCGAGAAGGAGGCGGACATATGGGACGTCGTGTAGTCGTCACC
LanArev	CCCCGCGGCCGCAAGCTTCCTTCGAATCATGCCGCGCTCCTCTCCGGGCG
LanBdir	CCCCCTCGAGTTCGAAGGAGGCGGACACATGACCGCAAGAGTCGTCATCACC
LanBrev	CCCCAAGCTTCCACTAGTCATCGGTCCGTGCCGCGCACGACC
LanCdir	CCCCCTCGAGACTAGTAAGGAGGCGGACACATGTCTCAGCCCGAGTTCACCGTCG
LanCrev	CCCCAAGCTTCCCCTAGGTCAGGCGGCGAGGGAGCCGGGCAGC
LanFdir	CCCCCTCGAGCCTAGGAAGGAGGCGGACACATGCACAGCACTCTGATCGTGGCG
LanFrev	CCCCAAGCTTCCCTTAAGTCACGAGGAAGCCGTCCAGTCGTAG
LanDdir	CCCCCTCGAGCTTAAGAAGGAGGCGGACACATGTCGCAGCAGGACCAGCGTGTCG
LanDrev	CCCCAAGCTTCCGCTAGCTCAGAAGTTGCCGAGTCCGCCGCAG
LanLdir	CCCCCTCGAGGCTAGCAAGGAGGCGGACACATGACGGTCCGTGAGGTCGAGCAC
LanLrev	CCCCAAGCTTCCCAATTGTCAGCGCTGCTGCTGCTCGGCGTAG
LanEdir	CCCCCTCGAGCAATTGAAGGAGGCGGACACATGGACGCTGCGGTGATCATCGC
LanErev	CCCCAAGCTTCCATGCATTCAGAAGGCGCGCCCGACCCGCG
Aur1Ddir	CCCCCTCGAGAAGGAGGCGGACATATGACCCGTCGGGTAGTGATCACG
Aur1Drev	CCCCGCGGCCGCAAGCTTCCTTCGAATCATGCGGTCCTCCTGTCGGGCC
Aur1Edir	CCCCCTCGAGTTCGAAGGAGGCGGACACATGAGCGCGAGGATCCTGGTCACC

Aur1Erev	CCCCAAGCTTCCACTAGTTCAGTCGACGGCGCGCACCACG
Aur1Fdir	CCCCCTCGAGACTAGTAAGGAGGCGGACACATGAAGGGATCCATTTTGTCCAAGC
Aur1Frev	CCCCAAGCTTCCCCTAGGCGTCGACCAGCGCGCTC
Aur1Cdir	CCCCCTCGAGCCTAGGAAGGAGGCGGACACATGACGCACAGCAACCTCATCG
Aur1Crev	CCCCAAGCTTCCCTTAAGTCATTCCGAGGCCGACCAGTCG
Aur1Gdir	CCCCCTCGAGCTTAAGAAGGAGGCGGACACATGTCGGATCAGGACAAGCGGG
Aur1Grev	CCCCAAGCTTCCGCTAGCTCAGAAGTTGCCGAGGCCGCCGC
Aur1Hdir	CCCCCTCGAGGCTAGCAAGGAGGCGGACACATGACGCAGCCCGGTCTGCGCG
Aur1Hrev	CCCCAAGCTTCCCAATTGTCAGTCCTTCTTCCCGCCCCGGG
Aur1Adir	CCCCCTCGAGCAATTGAAGGAGGCGGACACATGGACGCCCCAGTGATCGTCGTCG
Aur1Arev	CCCCAAGCTTCCATGCATTCAGGCGGGGCCGAACCAGCGC
Aur2Adir	CCCCCTCGAGAAGGAGGCGGACATATGAGCCGACGCGTCGTCATCACC
Aur2Brev	CCCCAAGCTTCCCCTAGGTTAATGCGCGGCGCGGACCACC
Aur1Ldir	CCCCCTCGAGCAATTGAAGGAGGTTGTTCATGACGGCCACCACGCTCCTCC
Aur1Mrev	CCCCAAGCTTCCCAATTGTCAGACTGTGGCGGCGGTGAGC

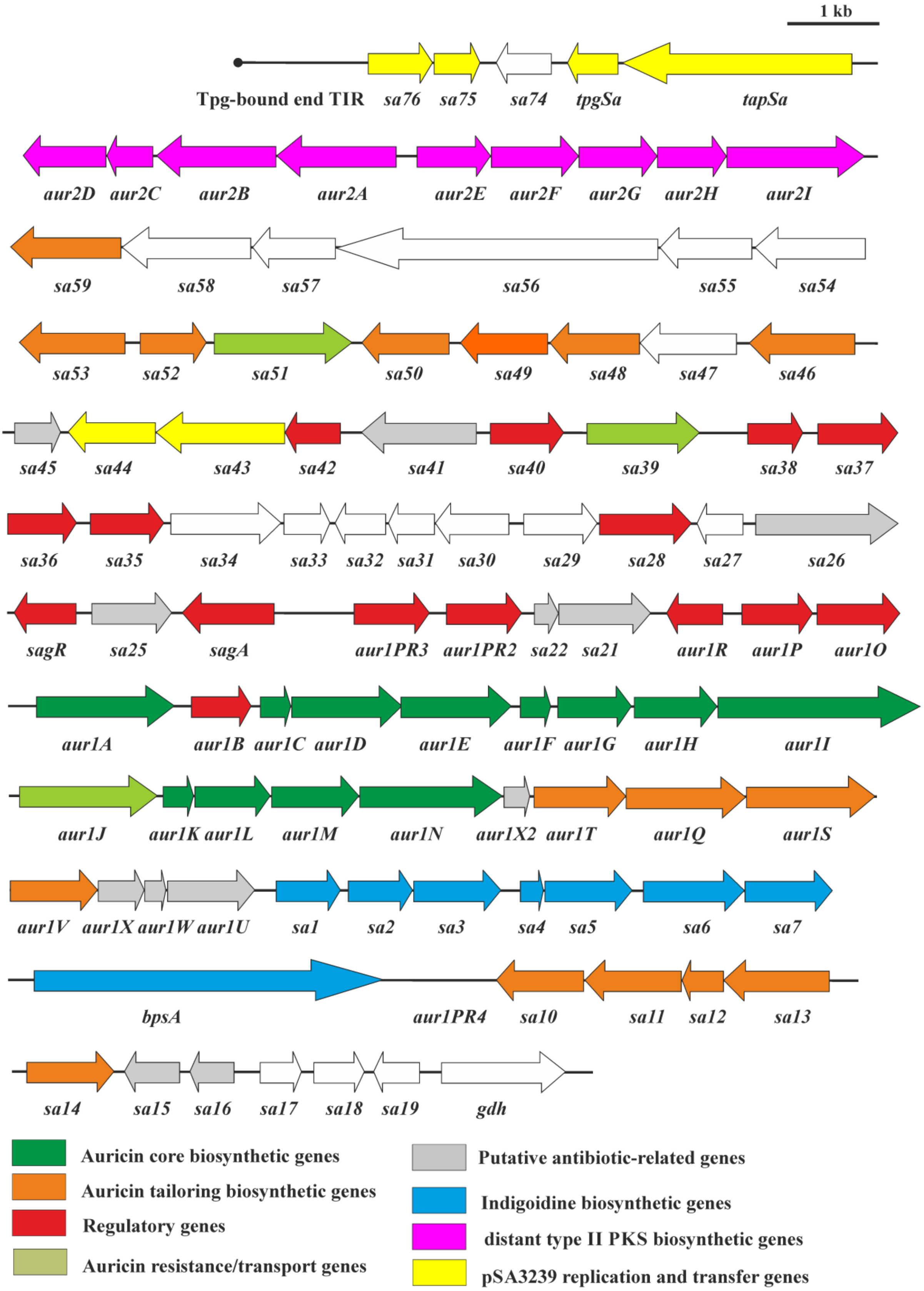

Fig. S1 Genetic organization of the *aur1* BGC for auricin and its flanking regions. Arrows indicates the position and direction of expression of indivilual genes. Details of the genes and their products are desribed in Genbank Acc. No. KJ396772.

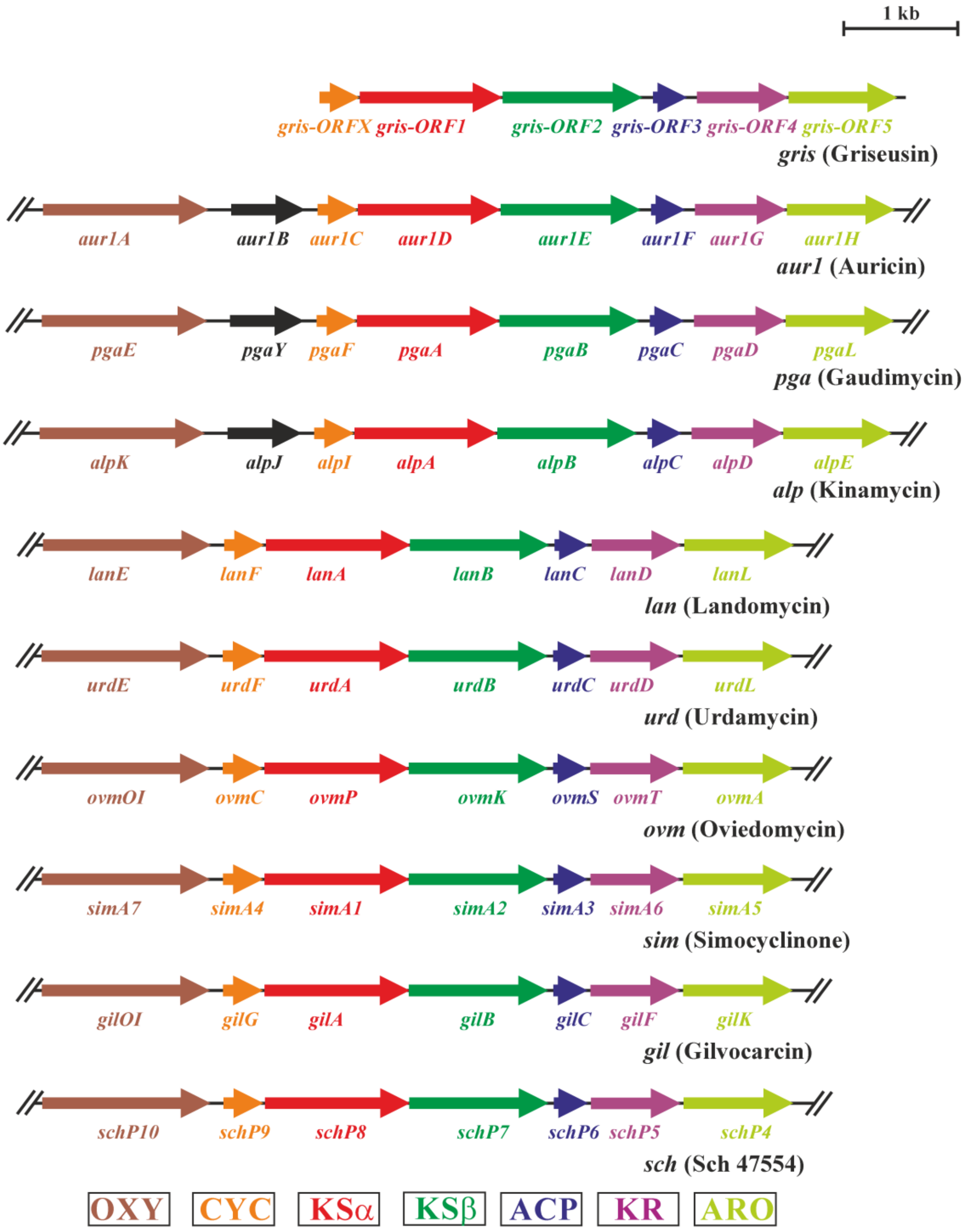

Fig. S2 Comparison of auricin *aur1* BGC core gene organization from *S. lavendulae* subsp. *lavendulae* CCM 3239 (Kormanec et al. 2014) with incomplete griseusin BGC from *S. griseus* K-63 (Yu et al. 1994) and other angucycline BGCs. Homologous genes are indicated by arrows with the same colour. BGC accession numbers are: *Streptomyces* sp. PGA64 gaudimycin (*pga*) BGC (AY034378), *S. ambofaciens* ATCC 23877 kinamycin (*alp*) BGC (AY338477), *S. cyanogenus* S136 landomycin (*lan*) BGC (AF080235), *S. fradiae* Tu2717 urdamycin (*urd*) BGC (X87093), *S. antibioticus* ATCC 11891 oviedomycin (*ovm*) BGC (AJ632203), *S. antibioticus* Tu6040 simocyclinone (*sim*) BGC (AF324838), *S. griseoflavus* Goe 3592 gilvocarcin (*gil*) BGC (AY233211), *Streptomyces* sp. SCC-2136 angucyclines Sch 47554 and Sch 47555 (*sch*) BGC (AJ628018). The conserved central regions encode oxygenase (OXY), angucycline-specific cyclase (CYC), ketosynthase α (KSα), ketosynthase β (KSβ), acyl carrier protein (ACP), ketoreductase (KR), and aromatase (ARO). In three BGCs, this region is interrupted by a gene (*aur1B*, *pgaY*, *alpJ*) encoding a conserved TetR family regulator.

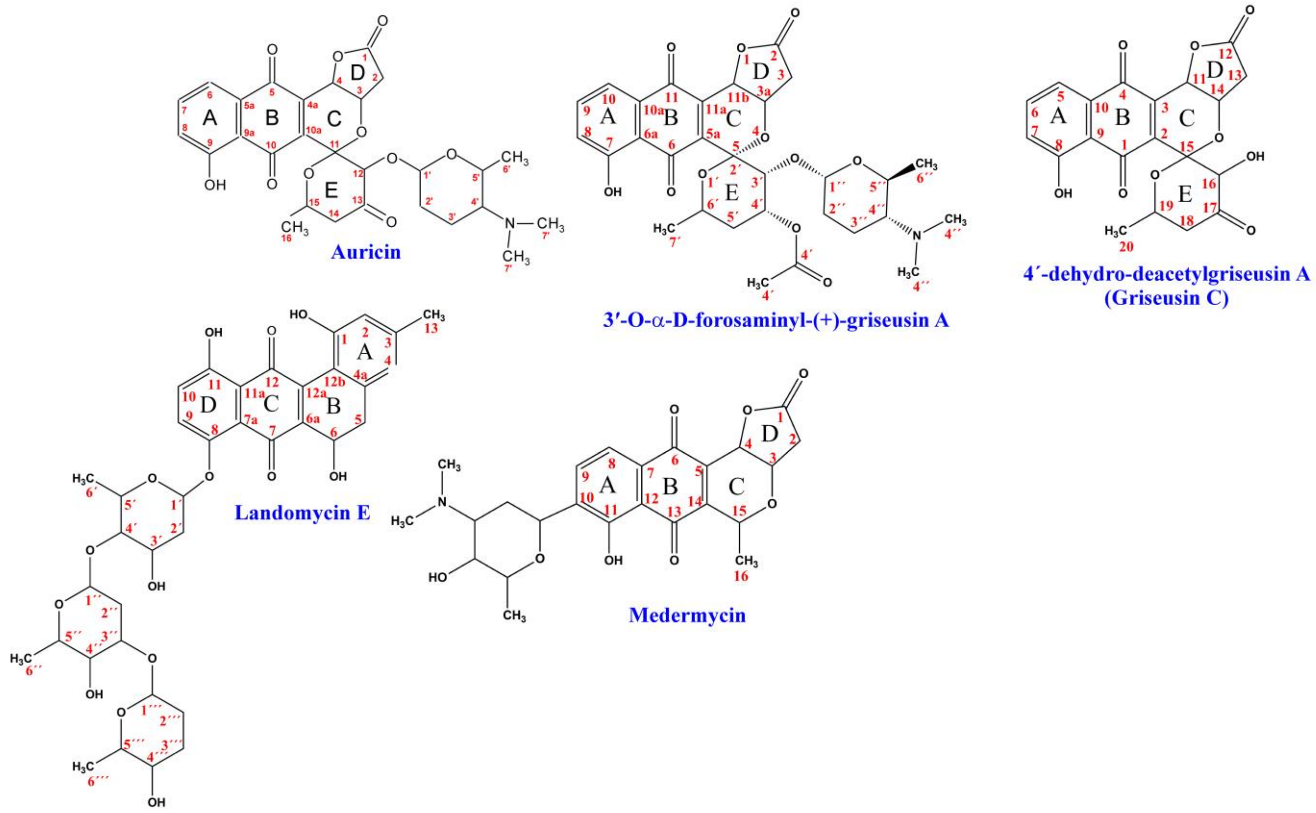

Fig. S3. Structure of auricin, 3´-O-α-D-forosaminyl-(+)-griseusin A, 4´-dehydro-deacetylgriseusin A (griseusin C), and landomycin E (angucycline group), and medermycin (pyranonaphthoquinone group). Numbering of positions is according to the published data for each compound.

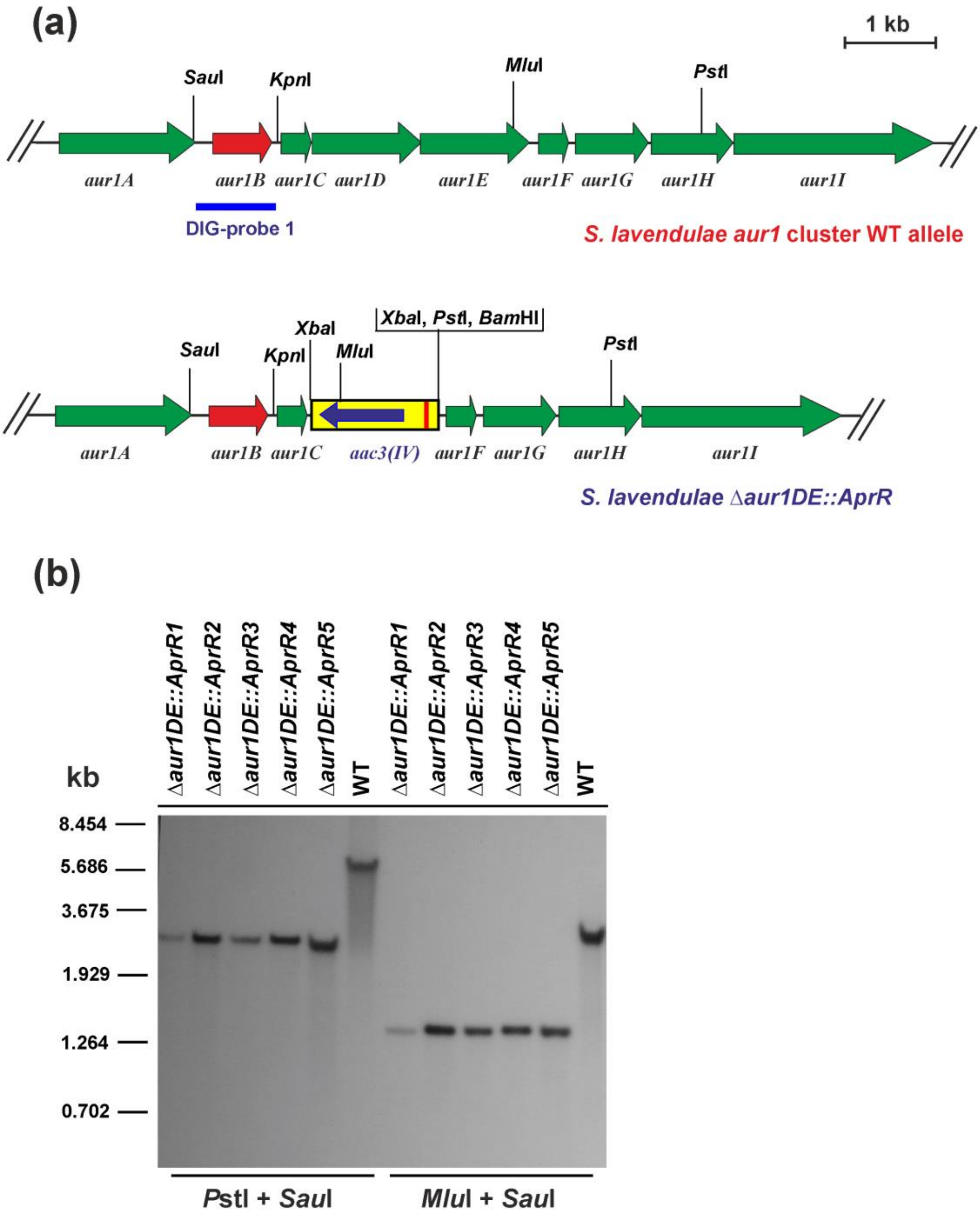

Fig. S4 a Genetic organization of the *aur1* BGC around the *aur1DE* genes in wild-type *S. lavendulae* subsp. *lavendulae* CCM 3239 (Kormanec et al. 2014; Matulova et al. 2019) and the disrupted strain *S. lavendulae Δaur1DE::AprR*. Coloured arrows indicate individual genes; green correspond to the *aur1* BGC and red to the regulatory gene. The yellow box with the blue arrow indicates the AprR *aac3(IV)* gene with the *oriT* origin of transfer (red column) from pIJ773 (Gust et al. 2003). The blue bar below the maps indicates the position of the probe 1 used for Southern hybridization analysis. Relevant restriction sites are included. (**b**) Southern blot hybridization analysis of five *S. lavendulae* Δ*aur1DE::AprR* clones and wild-type *S. lavendulae* subsp. *lavendulae* CCM 3239 (WT) as a control. 1 µg of DNA from the respective strain was digested with the indicated restriction endonucleases and separated by electrophoresis in a 0.8% (w/v) agarose gel. After transfer to a Hybond N membrane, hybridization was performed according to the standard DIG protocol as desribed in materials and methods using DIG-labelled probe 1 covering the *aur1B* gene. *Bst*EII-digested lambda DNA was used as a size standard.

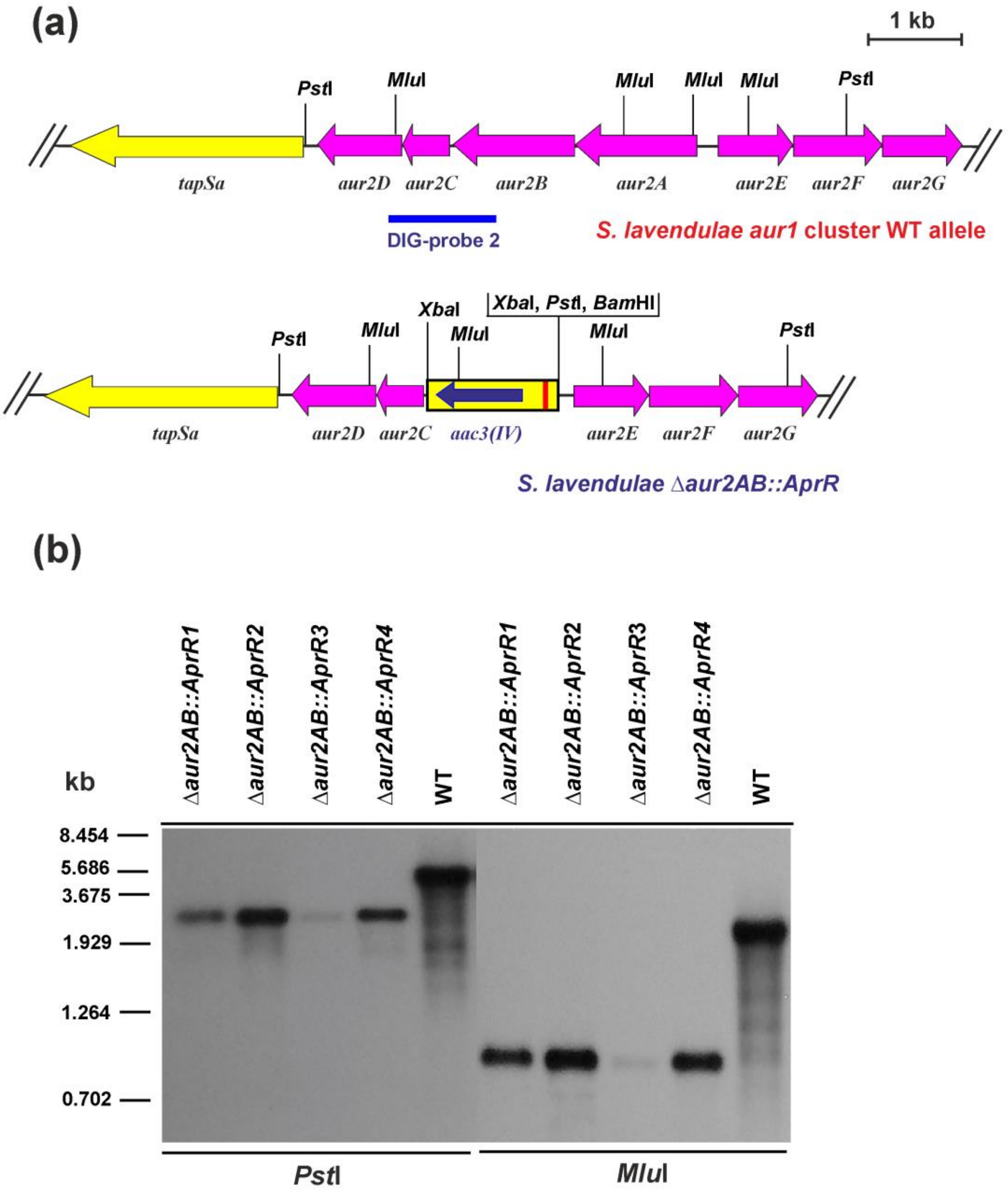

Fig. S5 a Genetic organization of the type II PKS BGC *aur2* around two translationally-coupled genes *aur2A* and *aur2B* encoding KSα and KSβ in wild-type *S. lavendulae* subsp. *lavendulae* CCM 3239 (Kormanec et al. 2014; Matulova et al. 2019) and the disrupted strain *S. lavendulae Δaur2AB::AprR*. Coloured arrows indicate individual genes; purple correspond to the *aur2* BGC and yellow to the pSA3239 replication gene. The yellow box with the blue arrow indicates the AprR *aac3(IV)* gene with the *oriT* origin of transfer (red column) from pIJ773 (Gust et al. 2003). The blue bar below the maps indicates the position of the probe 2 used for Southern hybridization analysis. Relevant restriction sites are included. (**b**) Southern blot hybridization analysis of four *S. lavendulae* Δ*aur2AB::AprR* clones and wild-type *S. lavendulae* subsp. *lavendulae* CCM 3239 (WT) as a control. 1 μg of DNA from the respective strain was digested with the indicated restriction endonucleases and separated by electrophoresis in a 0.8% (w/v) agarose gel. After transfer to a Hybond N membrane, hybridization was performed according to the standard DIG protocol as desribed in materials and methods using DIG-labelled probe 2 covering the *aur2BCD* gene region. *Bst*EII-digested lambda DNA was used as a size standard.

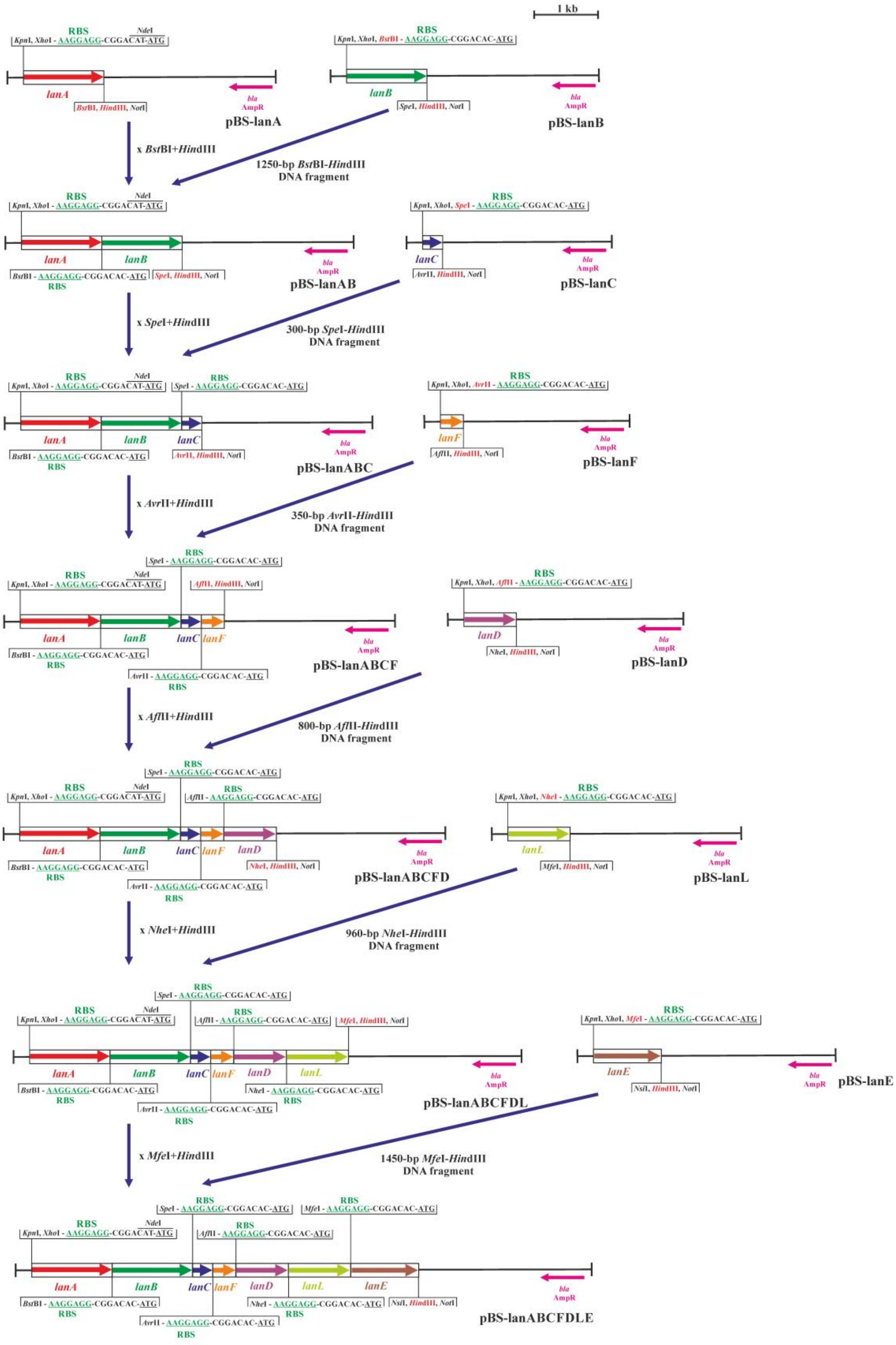

Fig. S6 Cloning strategy to create the artificial operon *lanABCFDLE*. Each individual *lan* gene was PCR amplified together with a strong RBS site and cloned into pBluescript II SK to obtain plasmids pBS-lanA, pBS-lanB, pBS-lanC, pBS-lanF, pBS-lanD, pBS-lanL, and pBS-lanE. Subsequently, the genes were inserted in the final operon according to the scheme to obtain the final plasmid pBS-lanABCFDLE. Sequences upstream of individual genes are shown with RBS from pMU1s* (Craney et al. 2007) in green and the ATG codon underlined. The relevant restriction sites are indicated.

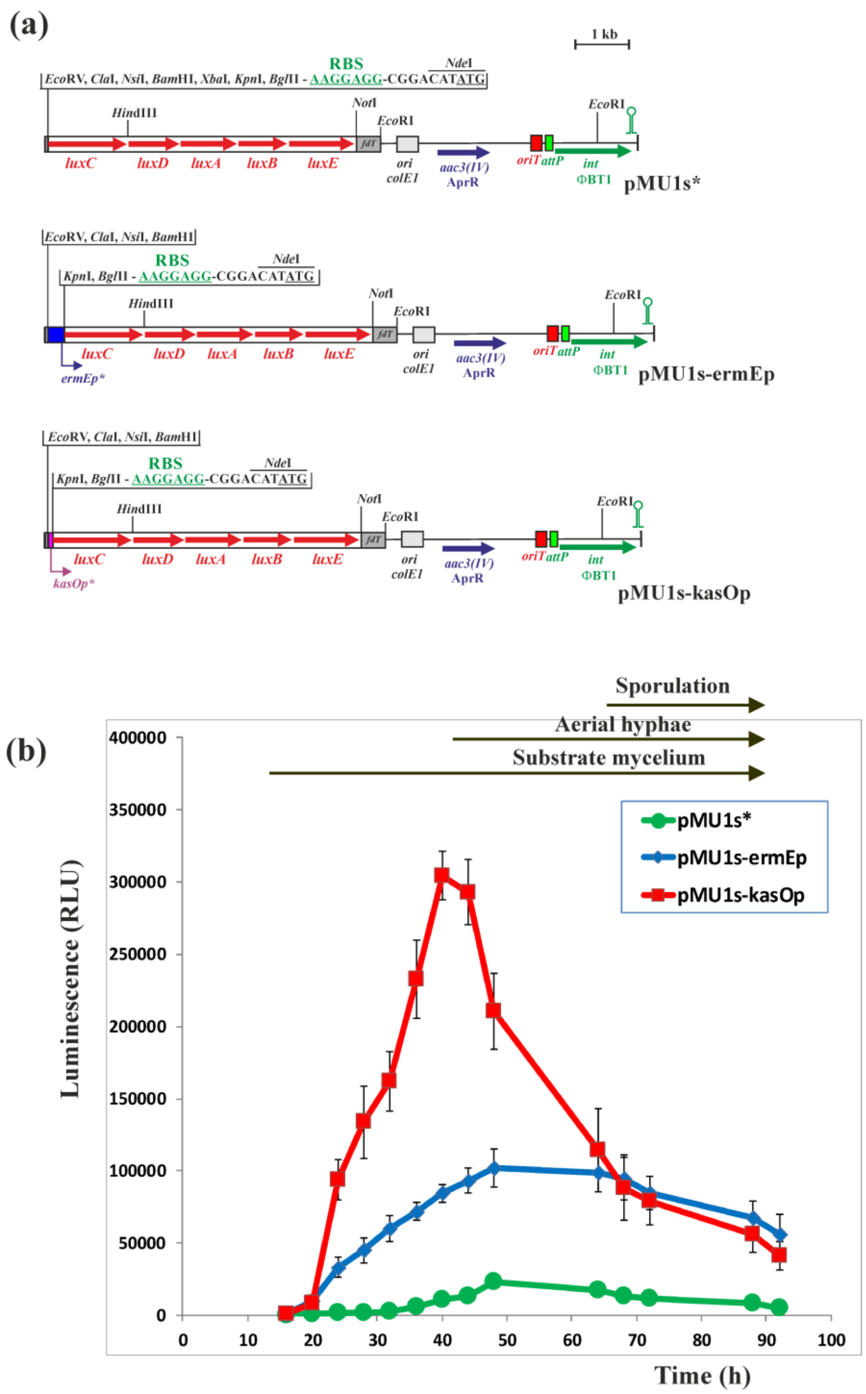

Fig. S7 a Schemes of the *luxCDABE* reporter PhiBT1 phage integration plasmid pMU1s* (Craney et al. 2007) and recombinant plasmids containing the *ermEp** or *kasOp** promoter. Arrows indicate the position and orientation of individual genes, and bent arrows indicate the position of promoters. The plasmid backbone contains the AprR *aac3(IV)* gene (blue arrow), the *oriT* origin of transfer (red bar), the *phiBT1int* integrase gene (green arrow) together with the *attP* site (green bar), the *fdT* terminator (dark grey box), and the *E. coli* origin of replication *ColE1* (light grey box) from plasmid pMU1s* (Craney et al. 2007). Only relevant restriction nuclease sites are shown. **b** The luciferase activity of the *luxCDABE* operon after fusion with the corresponding promoters. The plasmids were introduced into *S. coelicolor* M1146 by conjugation and the luminescence of eight clones from each construct was determined in relative luminescence units (RLU) after growth and differentiation on solid Bennet medium in 96-well plates at the indicated time points. Each time point represents the mean, and the error bar indicates the standard deviation from the mean. Black arrows above the graph indicate developmental stages.

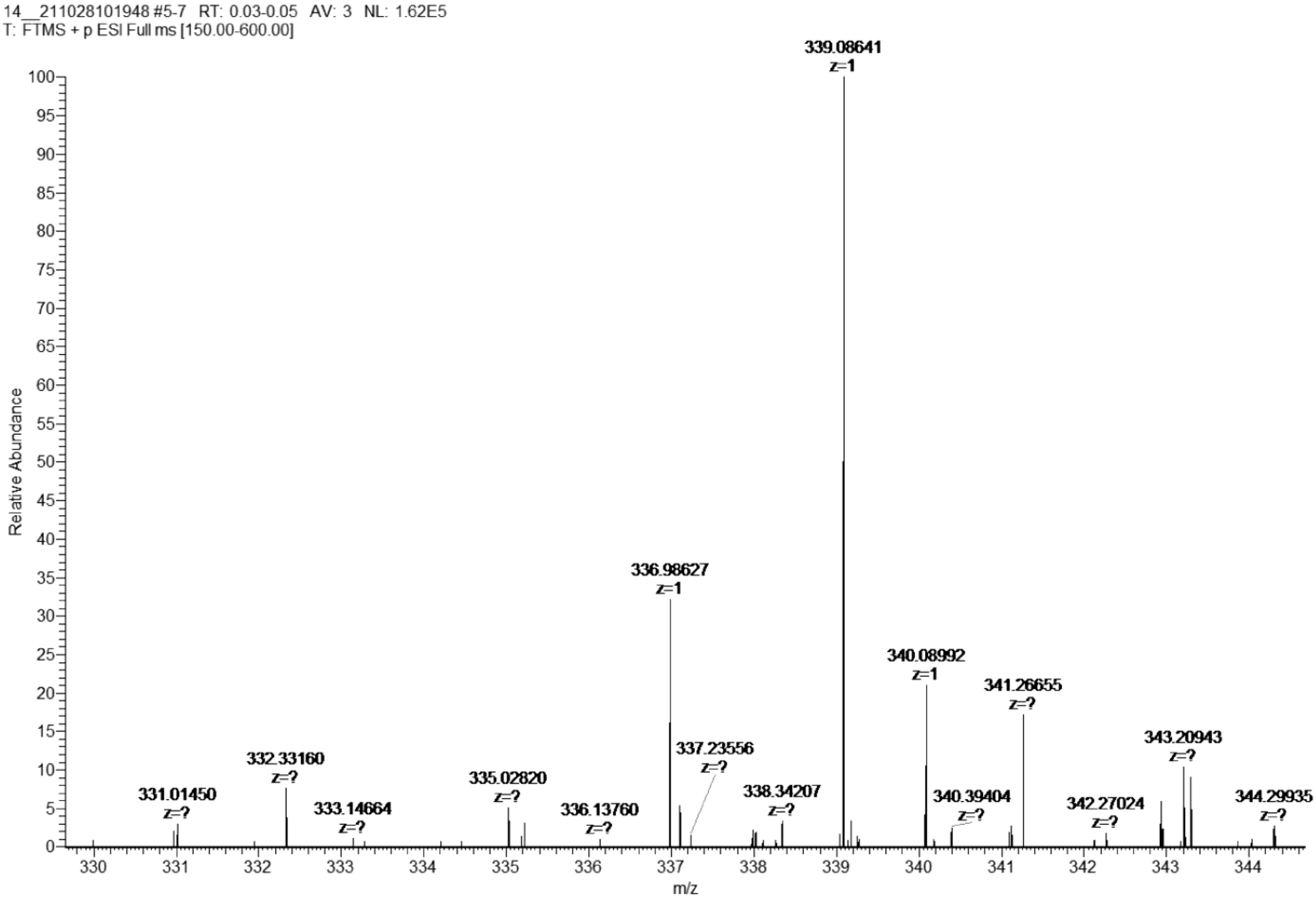

Fig. S8 High resolution ESI MS spectrum in positive mode for the rabelomycin peak (*m/z*=339.08641 [M=H]+, calculated mass is 339.08631).

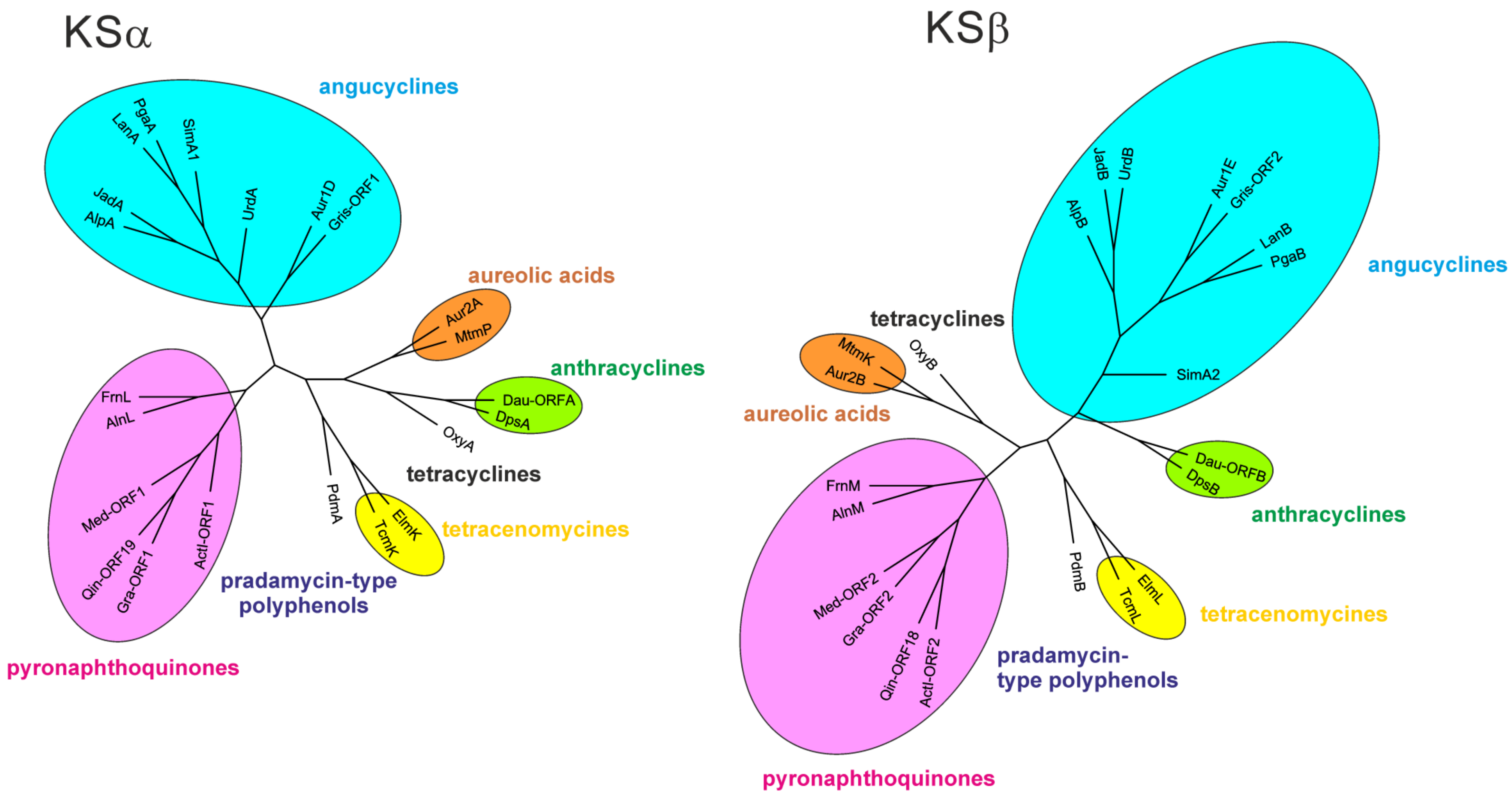

Fig. S9 Phylogenetic trees of selected ketosynthase α (KSα) and ketosynthase β (KSβ), which represent basic groups of aromatic polyketides. The Neighbour Joining method (Saiton and Nei 1987) was used to construct these trees based on the comparison of KSα (Aur1D, Aur2A) and KSβ (Aur1E, Aur2B) *from S. lavendulae* subsp. *lavendulae* CCM 3239 with the selected KSα and KSβ from the basic groups of aromatic polyketides. Protein alignments and their desriptions are shown in supplementary figures S8 and S9 below.

```
CLUSTAL X (1.81) multiple sequence alignment
Aur2A           -------------------------------------------MSRRVVITGIGVVAPGG
MtmP            -------------------------------------------MNRRVVITGIGVVAPGA
Gra-ORF1        -------------------------------------------VTRRVVITGVGVRAPGG
Qin-ORF19       -------------------------------------------MTRRVVITGIGVRAPGG
Med-ORF1        -------------------------------------------MTRRVVVTGLGVRAPGG
ActI-ORF1       VPLDAAPVDPASRGPVSAFEPPSSHGADDDDDHRTNASKELFGLKRRVVITGVGVRAPGG
AlnL            -------------------------------------------VNRSVAITGIGVVAPGG
FrnL            -------------------------------------------VNRQVAVTGIGVVAPGG
AlpA            ------------------------------------------LSRRRVVITGIEVIAPGG
JadA            ------------------------------------------VTARRVVITGIEVLAPGG
LanA            -------------------------------------------MGRRVVVTGIGVLAPGG
PgaA            -------------------------------------------VSRRVVITGVGVLAPGG
SimA1           -------------------------------------------VRRRVVITGVGVMAPGG
UrdA            ---------------------------------------VSGAHSRRVVITGIGVTAPGG
Aur1D           -------------------------------------------MTRRVVITGIGVLAPGG
Gris-ORF1       -------------------------------------------VERRAVITGIGVCAPGG
ElmK            ---------------------------------------MTG---RQVVITGIGVRAPGG
TcmK            ---------------------------------------MTRHAEKRVVITGIGVRAPGG
PdmA            ------------------------------------VSRPQGGGPRRVAITGMGVVAPGG
Dau-ORFA        -------------------------------------------VNRRVVITGMGVVAPGA
DpsA            -------------------------------------------VNRRIVITGIGVVAPGA
OxyA            --------------------------------------MSKIHDARRVVITGIGVVAPGD
                                                             :  .:**: * ***

Aur2A           VGTKEFWSLLTAGKTATRAISLFDASPFRSRIAAEADFNPLDHGFTAEEAMRLDRAAQFA
MtmP            VGVEAFWEQLTAGRTATRTISLFDASAFRSRIAAEVDFDAARHGFGPAEAERLDRATQFA
Gra-ORF1        SGTKEFWDLLTAGRTATRPISFFDASPFRSRIAGEIDFDAVAEGFSPREVRRMDRATQFA
Qin-ORF19       SGTKEFWDLLTAGRTATRRISFFDAAAFRSQVAAEADFFPEAEGFSPREIRRMDRATQFA
Med-ORF1        SGARQFWDLLSSGRTATRSITSFDASACRSQVAGEIDFDPVAEGLSPREIRRMDRAAQFA
ActI-ORF1       NGTRQFWELLTSGRTATRRISFFDPSPYRSQVAAEADFDPVAEGFGPRELDRMDRASQFA
AlnL            VGKKAFWDLLVSGRTATRTISFFDPSRFRSQVAAEVDFDPQRSGLSPREARRLDRAAQFA
FrnL            IGRKPYWEQLTSGRTATRAISFFDASPFRSRIAAEVDFDPAAAGLSPREVRRMDRAAQFA
AlpA            VGRENFWNLLSSGRTATRGITFFDPAPFRSRVAAEADFDPFAHGLSPQEVRRLDRAAQFA
JadA            TGSKAFWNLLSEGRTATRGITFFDPTPFRSRVAAEIDFDPEAHGLSPQEIRRMDRAAQFA
LanA            IGAENFWSLLSEGRTATRGITFFDPSSFRSQIAAEADFDAERSGLSPQEIRRMDRAAQFG
PgaA            IGAKNFWSLLSEGRTATRGITFFDPSSFRSRVAAEADFDPEMHGLTPQEIRRMDRAAQFA
SimA1           IGVKNFWSLLSEGRTATRGITFFDPAPFRSRVAAEIDFFPEKHGLGPQKIRRMDRAAQLA
UrdA            VGSKNFWSLLSDGRTATRRISFFDPSPFRSQVAAEADFDAELLGLSPQEIRRMDRAAQFA
Aur1D           IGTTAFWNLLSSGRTATRGITLFDPAPFRSRVAAEVDFHPERHGLTSLEVRRMDRAAQFG
Gris-ORF1       TGVDSFWELMCDGRTATRGITLFDPSPYRSRVAAEIDFDPRAHGLTPQEIRRMDRAAQFG
ElmK            TGVKAFWDLLSSGRTATRRISSFDPSPYRSQIAAEIDFDPLGEGFTDRQIARWDRAVLLA
TcmK            AGTAAFWDLLTAGRTATRTISLFDAAPYRSRIAGEIDFDPIGEGLSPRQASTYDRATQLA
PdmA            SGRKAFWNLLTDGRTATRKISLFDPAGFRSRIAAECDFDPAAEGLTPREVRRMDRAAQLA
Dau-ORFA        IGIKSFWELLLSGTTATRAITTFDATPFRSRIAAECDFDPVAAGLSAEQARRLDRAGQFA
DpsA            VGTKPFWELLLSGTTATRAISTFDATPFRSRIAAECDFDPVAAGLSAEQARRLDRAGQFA
OxyA            VGTKPFWEMLTAGRTATRPISSFDASPFRSQVAAECDFDPAAEGLSQRQVRAWDRTMQFA
                 *   :*. :  * **** *: **.:  **::*.* ** .   *:   :    **:  :.

Aur2A           IVSASEAVRDSGIDTAELDPTRTGVTLGSAVGCTTGLDVEYNAVSRGGSTWQVDHTLAVP
MtmP            LVSAREAVADAGLD-GKTDPSRTGVALGSAVGCTTGLDTQYNVVSEGGSDWYVDHTRAVD
Gra-ORF1        VACTRDALADSGLDTGALDPSRIGVALGSAVASATSLENEYLVMSDSGREWLVDPAHLSP
Qin-ORF19       VACTREAVADSGLEFAGVDPHRVGVSLGSAVASATSLENEYLVMADKGREWLVDPDYLSP
Med-ORF1        VVCSREAVADSGLSFEGVRPERIGVSVGSAVAAAMSLEKEYRVLSDQGREWEVDPTYLTP
ActI-ORF1       VACAREAFAASGLDPDTLDPARVGVSLGSAVAAATSLEREYLLLSDSGRDWEVDAAWLSR
AlnL            VVSARECMADSGLEFVELDPHRTGVSVGSAVGGTTGLEREYLVLSDSGRLWEVDSDYVSP
FrnL            VVSARESLADSGLDVADLDPHRIGVSIGSAVGGTTSLEREYLALSDSGRQWELDLSYLSP
AlpA            VVASRGAVADSGLDVAALDPHRVGVTVGSAVGATMGLDEEYRVVSDGGRLEAVDHTYAVP
JadA            VVAAR-AVADSGIDLAAHDPYRVGVTVGSAVGATMGLDEEYRVVSDGGRLDLVDHAYAVP
LanA            VVTAREALADSGLDQAGLDPYRTGVTIGSAVGATMGLDEEYRVVSDGGRLDLVDHRYTPQ
PgaA            VVTAREALADSGLDLAGFDPHRTGVTIGSAVGATTGLDDEYRVVSDGGRLDLVDHTYAPP
SimA1           VIATREAVADSGLDLDATDPHRRGVTVGSAVGATTGLDQEYRVVSDDGRLGLVDHRYAPP
UrdA            VVTAREAVADSGLEFASLDPHRTGVTVGSAVGATMGLDQEYRTVSDSGRLDLVDHEYAVP
Aur1D           VVATREALADSGLDLAAFDPYRIGIAMGTGVGAISGLDTAYRVASDEGRIAQVDHMYAPG
```

```
Gris-ORF1       VVTAREAFADSGLDGAALDPFRTGVSLGTAIGAAGGLDAEYRVVSDDGRLELVDHRYATP
ElmK            VAAAREALAHSGLAPGALRPETVGVSVGSAVGCTTSLDTEYARVSHGGADWLVDHTLAVQ
TcmK            VVCAREALKDSGLDPAAVNPERIGVSIGTAVGCTTGLDREYARVSEGGSRWLVDHTLAVE
PdmA            VVSAREALADSGLVAGEGDPARFAVSLGSAVGCTMGLEDEYVVVSDQGRDWLVDHSYGVP
Dau-ORFA        LVAGQEALTDSGLRIGEDSAHRVGVCVGTAVGCTQKLESEYVALSAGGANWVVDPHRGAP
DpsA            LVAGQEALADSGLRIDEDSAHRVGVCVGTAVGCTQKLESEYVALSAGGAHWVVDPGRGSP
OxyA            YVAAREALADSGV-TGEADPLRTGVMAGTACGMTMSLDREYAVVSDEGRLWQVDDAHGVP
                      ..  :*:      .   .:  *:. .    *:  *   :  *    :*

Aur2A           HLFDYFVPSSMAAEVAWRVNAQGPVSMVSTGCTSGLDSIGHAV-ELIREGSADVMITGAT
MtmP            HLFDYFVPSSMAAEVAWSIGAQGPVALISTGCTSGLDSLGHAV-ALIREGSADVMVAGAT
Gra-ORF1        MMFDYLSPGVMPAEVAWAAGAEGPVTMVSDGCTSGLDSVGYAV-QGTREGSADVVVAGAA
Qin-ORF19       HMFDYLSPGVMPAEVAWAAGAEGPVTMVSDGCTSGLDAVGYGV-QLIREGSADVMIAGAS
Med-ORF1        HMFDHMVPSVLGAEVAWTVGAEGPVTVVSDGCTSGLDSVGYAC-RLIQEGSVDVMLAGAT
ActI-ORF1       HMFDYLVPSVMPAEVAWAVGAEGPVTMVSTGCTSGLDSVGNAV-RAIEEGSADVMFAGAA
AlnL            HLFDAFVPSSLAAEVAWTVGAEGPATVVSTGCTSGLDSVGYAR-DLIAEGTVDVMIAGAA
FrnL            HLYDAFTPSSLAREVAGVIGAEGPAAVVSTGCTSGIDSLGHAR-DLIAEGSADVVLAGGT
AlpA            HLYDYMVPSSFAAEVAWAVGAEGPSTVVSTGCTSGIDSVGYAV-ELVREGSADVVIAGSS
JadA            HLYDYMVPSSFSAEVAWAVGAEGPNTVVSTGCTSGLDSVGYARGELIREGSADVMIAGSS
LanA            HLYNHFVPSSFSAEVAWAVGAEGPNTVVSTGCTSGIDSVGQAV-ELIREGSVDVMIAGAT
PgaA            HLYNYFVPSSFSAEVAWAVGAEGPATVVSTGCTSGLDSVGHAT-ELIREGTADVMITGAT
SimA1           HLYGHFVPSSFAAEVAWASGSEGPSTVVSTGCTSGLDSLGHAV-ELIREGSADVMIAGAT
UrdA            HLYNYLVPSSFAAEVAWAVGAEGPATVVSTGCTSGLDAVGYAT-ELIREGSADVMVAGAA
Aur1D           HLYDHFVPSSFATEVAWAVGAEGPSTVVSTGCTSGIDSVAHAV-DLIREGSADIMITGAT
Gris-ORF1       HLYDLFVPSSFATEVAWAVGAEGPATVVSTGCTAGIDAVGHAV-EAIRDGSADVMVTGAV
ElmK            QLFDYFVPTSISREVAWEVGAEGPVTLVSTGCTSGLDAVGNGA-SLIRDGNADVVLAGAT
TcmK            QLFDYFVPTSICREVAWEAGAEGPVTVVSTGCTSGLDAVGYGT-ELIRDGRADVVVCGAT
PdmA            HLYRHLVPSSLAAEVAWAGGAEGPVTLISTGCTSGLDAVGHGA-RVIAEGSADVALAGAT
Dau-ORFA        ELYDYFVPSSLAAEVAWLAGAEGPVNIVSAGCTSGIDSIGYAC-ELIREGTVDVMLAGGV
DpsA            ELYDYFVPSSLAAEVAWLAGAEGPVNIVSAGCTSGIDSIGYAC-ELIREGTVDAMVAGGV
OxyA            YLYDYFVPSSMAAEIAWLAEAEGPAGVVSAGCTSGIDVLTHAA-DLVRDGAAEVMVAGAS
                 ::  : *  :  *:*    ::**  ::* ***:*:* :  .      :* .:  . *.

Aur2A           EAPISPITVACFDAIKATSPRNDDPGTASRPFDATRNGFVLGEGSAVLVLEDLESARKRG
MtmP            EAPISPITVACFDAIKATSPRNDDPATASRPFDRSRNGFVLGEGSAVLILESLENARRRD
Gra-ORF1        DTPVSPIVVACFDAIKATTPRNDDPAHASRPFDGTRNGFVLAEGAAMFVLEEYEAAQRRG
Qin-ORF19       DTPISPIVLACFDAIKATTTRNDDPEHASRPFDASRNGFVLAEGSAMFVLEEYEAAKARG
Med-ORF1        DTPLTPIVAACFDAIKATTPRNDDAEHASRPFDLSRNGFVLAEGAAMFVLEEYESAVRRG
ActI-ORF1       DTPITPIVVACFDAIRATTARNDDPEHASRPFDGTRDGFVLAEGAAMFVLEDYDSALARG
AlnL            DTPISPIAVSCFDAIKATTPRNDDPEHASRPFDRTRNGFVLAEGAAMFVLEELEHARARG
FrnL            DTPISPIAVACFDAIKATSPSNDDPAHASRPFDRERNGFVLAEGAAVLVLEELGHARARD
AlpA            DAPISPITMACFDAIKATTPRHDEPECASRPFDKTRNGFVLGEGCAFFVLEELDSARKRG
JadA            DAPISPITMACFDAIKATTNRYDDPAHASRPFDGTRNGFVLGEGAAVFVLEELESARARG
LanA            DAPISPITMACFDAIKATTPRNDEPEHASRPFDGTRNGFVLGEGSAVFVLEELTAARARG
PgaA            DAPISPITMACFDAIKATTPRNDDAAHASRPFDGTRNGFVLGEGSAVFVLEELESAKKRG
SimA1           DAPISPITMACFDAIKATTPRNDDPAHASRPFDRSRNGFVLGEGHAVLVLEELGAAQRRG
UrdA            DAPISPITVACFDAIKATTPRNEDPEHASRPFDGTRNGFVLGEGSAVFVLEELDSALRRG
Aur1D           DAPISPLTLACFDAIKATTALNEEPETASRPFDATRKGFVLGEGAAAFVLEELGAARRRG
Gris-ORF1       DAPITPLTMAAFDAIRATTSLNDEADTASRPFDASRKGFVLGEGAAVFVLEEAEHARRRG
ElmK            DAPISPITVACFDAIKATSPNNADPEHASRPFDRRRDGFVLGEGAAMFVLEEKEAALRRG
TcmK            DAPISPITVACFDAIKATSANNDDPAHASRPFDRNRDGFVLGEGSAVFVLEELSAARRRG
PdmA            DAPISPITVACFDAIRATSPNNDDPEHASRPFDRERNGFVLGEGAAVFVLEELEHARRRG
Dau-ORFA        DAPIAPITVACFDAIRVTSDHNDTPETLA-PFSRSRNGFVLGEGGAIVVLEEAEAAVRRG
DpsA            DAPIAPITVACFDAIRATSDHNDTPETASRPFSRSRNGFVLGEGGAIVVLEEAEAAVRRG
OxyA            DAAISPITVACFDAIKATTPRNDEPETASRPFDRTRNGFVLGEGAAFFVLEEYAHARRRG
                ::.::*:. :.****:.*:     .   : **.  *.****.** * .:**.   *  *.

Aur2A           AHVYAEISGFASRCNAYHMTGLRADGVEMAEAITVALDEARLDPSAVDYINAHGSGTKQN
MtmP            AHVYAEISGFASRCNAYHMTGLRPDGIEMAEAIRTALDEARLDPTAVDYVNAHGSGTKQN
Gra-ORF1        AHIYAEVGGYATRSQAYHMTGLKKDGREMAESIRAALDEARLDRTAVDYVNAHGSGTKQN
Qin-ORF19       AQIYAEVTGYATRCNAYHMTGLKKDGREMAEAIRAALDESRVDPTVVDYVNAHGSGTKQN
Med-ORF1        ARIYAEVTGYATRLNAHHMTGLKTDGREMAEAIRVALDESRIDPTAIDYVNAHGSGTKQN
ActI-ORF1       ARIHAEISGYATRCNAYHMTGLKADGREMAETIRVALDESRTDATDIDYINAHGSGTRQN
AlnL            AHVYGVIGGYATRCNAYHMTGLRPDGHEMAEAIRHSLDQARLNPDLVDYVNAHGSGTKQN
```

```
FrnL           AHVYALVSGYATRCNAYHMTGLTPHGREMAEAIRHALAESGTDPAAVDYVNAHGSGTKQN
AlpA           AHIYAEIAGYSTRSNAYHMTGLRPDGAEMAEAIDLALAEARLNPQAIDYVNAHGSGTKQN
JadA           AHIYAEIAGYATRSNAYHMTGLRPDGAEMAEAIRVALDEARMNPTEIDYINAHGSGTKQN
LanA           AHIYAEIAGYASRCNAFHMTGLRPDGREMGEAIRVALDEARINPEAIDYINAHGSGTKQN
PgaA           AHIYAEIAGYASRCNAFHMTGLRPDGREMSEAIDVALGEARMNPDRIDYINAHGSGTKQN
SimA1          ARIYAEIGGYASRCNAFHMTGLRPDGREMAEAIRVALDEARLNPEDIDYINAHGSGTKQN
UrdA           AHVYAEIAGYATRSNAFHMTGLRPDGREMAEAIRIALDEARLNPEDIDYVNAHGSGTKQN
Aur1D          AHVYAEIAGYASRSSAFHMTGLRPDGREMAEAITVALDEARLDPGDLDYVNAHGSGTRQN
Gris-ORF1      AYVYAEIAGYASRGNAFHMTGLRSDGAELAAAIRAALDEARLDASAVDYVNAHGSGTRQN
ElmK           APVLAEVAGFATRANAYHMTGLRSDGREMAAAIDAALLAAGRGPADVDYINAHGSGTRQN
TcmK           AHAYAEVRGFATRSNAFHMTGLKPDGREMAEAITAALDQARRTGDDLHYINAHGSGTRQN
PdmA           AHVYCEVAGYATRGNAYHMTGLKPDGREMAEAIRVAMDAARVAPADLDYINAHGSGTKQN
Dau-ORFA       ARIYAEIGGYASRGNAYHMTGLRADGAEMAAAITAALDEARRDPSDVDYVNAHGTATRQN
DpsA           ARIYAEIGGYASRGNAYHMTGLRADGAEMAAAITAALDEARRDPSDVDYVNAHGTATKQN
OxyA           ARAYAEIAGYAGRCNAYSMTGLRSDGRELAEAVSRALDIARVDPSEVDYVNAHGSATKQN
               *     : *:: * .*. ****  .* *:. ::  ::  :      :.*:****:.*:**

Aur2A          DRHETAAFKRSLGQRAYEVPVSSIKSMIGHSLGAIGSLEVAASALAIEYNTVPPTANLHT
MtmP           DRHETAAFKRSLKDHAYRVPVSSIKSMIGHSLGAIGSLEVAASALAIEHDTVPPTANLHD
Gra-ORF1       DRHETAAFKRSLGEHAYAVPVSSIKSMGGHSLGAIGSIEIAASVLAIEHNVVPPTANLHT
Qin-ORF19      DRHETAAFKIALGQHAYDVPVSSIKSMVGHSLGAIGSIEIAACALAIKHNVVPPTANLHT
Med-ORF1       DRHETAAFKRSLGDHAYAVPVSSIKSMVGHSLGAIGSIEIAASLLALTHQVVPPTANLHT
ActI-ORF1      DRHETAAYKRALGEHARRTPVSSIKSMVGHSLGAIGSLEIAACVLALEHGVVPPTANLRT
AlnL           DRHETAAFKETLGQHAYEVPISSIKSMVGHSLGAIGSIEIAACALAMENGAVPPTANLHE
FrnL           DRHETAAFKATLGERARSVPVSSIKSMIGHSLGAIGSLEIAAWALAMEYGVVPPTANLDT
AlpA           DRHETAAFKRSLGEHAFRTPVSSIKSMVGHSLGAIGSIEIAASALAMEYDVVPPTANLHT
JadA           DRHETAAFKKSLGDHAYRTPVSSIKSMVGHSLGAIGSIEIAASALAMEHNVVPPTGNLHT
LanA           DRHETAAFKLSLGEHAYRTPISSIKSMVGHSLGAIGSIEIAASLLAMENHVVPPTANLHT
PgaA           DRHETAAFKLSLGDHAYRTPVSSIKSMVGHSLGAIGSIEIAASVLAMKNHVVPPTANLHT
SimA1          DRHETAAFKRSLGDHAYRTPVSSIKSMVGHSLGAIGSIEIAACVLAMAESVIPPTANLHD
UrdA           DRHETAAFKRSLGDHAYAVPVSSIKSMVGHSLGAIGSIEIAASALAMEHGVVPPTANLHT
Aur1D          DLHETAAYKLALGPHAHRTPVSSVKSMIGHSLGSVGSMEIAASVLAMEHHVVPPTANLRT
Gris-ORF1      DRHETVALKSALGQHAHRVPVSAIKSMVGHSLGAIGSIEIAACALAMRHHVIPPTANLHT
ElmK           DRHETAAFKRSFGERAYAIPVSSIKSMIGHSLGAIGSLELAACVLAMESDLIPPTANYAE
TcmK           DRHETAAFKRSLGQRAYDVPVSSIKSMIGHSLGAIGSLELAACALAIEHGVIPPTANYEE
PdmA           DRHETAAFKRSLGERAYELPVSSIKSMVGHSLGAIGSIELAACALAIEHGVVPPTANLHN
Dau-ORFA       DRHETSAFKRSLGDHAYRVPISSVKSMIGHSLGAAGSLEVAATALAVEYGAIPPTANLHD
DpsA           DRHETSAFKRSLGEHAYRVPISSIKSMIGHSLGAVGSLEVAATALAVEYGVIPPTANLHD
OxyA           DLHETAAFKRSLGPHAYSVPISSIKSMIGHSLGAICALEVAASALRIEHGVIPPTANLRE
               * *** * * ::  :*   *:*::*** *****:  ::*:**  * :    :***.*

Aur2A          PDPSCDLDYTPLTAREQRTDAVLSVGSGFGGFQSAMILTRPRLGDAA--
MtmP           RDPACDLDYTPITAREQRTDTVLSVGSGFGGFQSAMVLTAPGLREAA--
Gra-ORF1       PDPECDLDYVPLTAREQRVDTVLTVGSGFGGFQSAMVLHRPEEAA----
Qin-ORF19      PDPECDLDYVPLTAREQRVDSVLTVGSGFGGFQSAMILRAPEGVKA---
Med-ORF1       ADPECDLRYVPLTAREAPVRSVLTVGSGFGGFQSAMVLRRPEEAAA---
ActI-ORF1      SDPECDLDYVPLEARERKLRSVLTVGSGFGGFQSAMVLRDAETAGAAA-
AlnL           PDPECDLDYVPNEAREHGVDAVLSVGSGFGGFQSAMVITREETTR----
FrnL           PDPECDLDYVPHEARETRVDRVLSVGSGFGGFQSAMVLTRDTGARLPTA
AlpA           ADPECDLDYVPLVARDQLIDAVLTVGSGFGGFQSAMVLASPERSLV---
JadA           PDPECDLDYVR-SCREQLTDSVLTVGSGFGGFQSAMVLARPERKIA---
LanA           PDPECDLDYVPLVAREHTTDTVLTVGSGFGGFQSAMVLARPERSAA---
PgaA           PDPECDLDYVPLTAREQRTDTVLTVGSGFGGFQSAMVLARPERNAA---
SimA1          PEPECDLDYVPITAREARLDRVLSVGSGFGGFQSAMVLTRPERNTA---
UrdA           PDPECDLDYVPRTARDWKTDAVLSVGSGFGGFQSAIVLARPDRRTA---
Aur1D          PDPECDLDYVPLVARDHRTDAVLTVSSGFGGFQSAMVLARPDRRTA---
Gris-ORF1      PDPECDLDYVPLTARAQRTDAVLTIGSGFGGFQSAMVLARPDRSAA---
ElmK           PDPECDLDYVPNTAREARLDTVVSVGSGFGGFQSAAVLTRPEGRPR---
TcmK           PDPECDLDYVPNVAREQRVDTVLSVGSGFGGFQSAAVLARPKETRS---
PdmA           ADPECDLDYVPLVAREGRIRTVLSVGSGFGGFQSATVLREAA-------
Dau-ORFA       PDPELDLDYVPLTAREKRVRHALTVGSGFGGFQSAMLLSRPER------
DpsA           PDPELDLDYVPLTAREKRVRHALTVGSGFGGFQSAMLLSRLER------
OxyA           PDPDCDLDYVPLVAREAEVSTVVSVASGFGGFQSAIVLTEPGRQR----
                :*  ** *.   .*      .:::.********* ::
```

Fig S10. Comparison of the amino acid sequence of KSα (Aur1D, Aur2A) *from S. lavendulae* subsp. *lavendulae* CCM 3239 with selected KSα proteins from the basic groups of aromatic polyketides. Protein sequences (and corresponding accession numbers) are as follows: auricin Aur2A (AIE41912), mithramycin MtmP (CAA61989), granaticin Gra-ORF1 (CAA09653), qinimycin Qin-ORF19 (WP_058047374), medermycin Med-ORF1 (BAC79044), actinorhodin ActI-ORF1 (CAC44200), alnumycin AlnL (ACI88861), frenolicin FrnL (AAC18107), kinamycin AlpA (AAR30152), jadomycin JadA (AAB36562), landomycin LanA (AAD13536), gaudimycin PgaA (AAK57525), simocyclinone SimA1 (AAK06784), urdamycin UrdA (CAA60569), auricin Aur1D (AAX57191), griseusin Gris-ORF1 (CAA54860), elloramycin ElmK (CAP12600), tetracenomycin TcmK (AAA67515), pradimicin PdmA (ABM21747), daunorubicin Dau-ORFA (AAA87618), doxorubicin DpsA (AAA65206), oxytetracycline OxyA (AAZ78325).

```
CLUSTAL X (1.81) multiple sequence alignment
ElmL            ---------------VTGSDTEDGSTGWITGLGVVAPNGIGAEEYWKATLEGRSGLRTIT
TcmL            --------------------MSAPAPVVVTGLGIVAPNGTGTEEYWAATLAGKSGIDVIQ
PdmB            ---------------------------------VVAPTGIGVEEHWAATLRGVPVIGPLT
JadB            ---------------MSA-------SVVVTGLGVAAPNGLGREDFWASTLGGKSGIGPLT
UrdB            ------------VNTGAV-------EVAVTGLGVVAPNGLGTDAYWAATRKGTSGIARIS
AlpB            ---------------MTA-------SVVVTGLGVTAPNGLGLKDYWAATLGGKHGIGRIT
Aur1E           ---------------MSA-------RILVTGIGVAAPSGLGVEDFWSVTRIGKNAIGPVT
Gris-ORF2       ---------------VSAPGGGDRGRTLITGMGLATPHGVDVEDFWAATRVGKNAIGPVT
LanB            ---------------MTA-------RVVITGIGIAAPNGFGVEDYWAATRVGKSAIGRIT
PgaB            ---------------MST-------RTVITGIGVATPNGLGVDEFWAATRVGKNAIARVT
SimA2           ---------------MTT-------SVVVTGLGVAAPNGLGTADYWAATREGRSGIGRVT
Dau-ORFB        ---------------------------VVTGLGIVAPNGLGVGAIWDAVLNGRNGIGPLR
DpsB            MTGTAARTASSQLHASPAGRRGLRGRAVVTGLGIVAPNGLGVGAYWDAVLNGRNGIGPLR
ActI-ORF2       ----------------MS--------VLITGVGVVAPNGLGLAPYWSAVLDGRHGLGPVT
Qin-ORF18       ----------------MTSKDGLNGRTVITGIGVTAPNGLGTEAFWKAVLAGHTGIGPVT
Gra-ORF2        --------------VSTPDRR----RAVVTGLSVAAPGGLGTERYWKSLLTGENGIAELS
Med-ORF2        ----------------MSDR------ALITGIGVVAPNGLGVKEYWNATLEGRGGIAPLT
AlnM            ------MTLATP-AAQETPERTGRPTAVITGIGVAAPNGLGTEQWWQSTLQGTSGIGPVV
FrnM            ------MTTAPSRTAQGAPPGAALP-PVFTGIGVAAPNGLGTEEWWAATLRGEHGLRPVT
Aur2B           -----MTTETAARPGGTPLSP--TAAPVVTGIGVTAPNGLGTEAWWAAVLRGESGIRPVG
MtmK            -----MSADASQ--------------AVITGIGVAAPNGLSVKAWWDAVLRGESGIRRLS
OxyB            -----MTGQLAPAPETGTGRPGGSVRPVVTGLGVVAPNGLGTERYWAATLRGDSGIGRIT
                                                 :.:* * .    *     *   :  :

ElmL            GFDAGQYPVRVAGEVGTFADEPSLSGRILPQTDRMTRY-ALVASDWALADSGVRT-DEHD
TcmL            RFDPHGYPVRVGGEVLAFDAAAHLPGRLLPQTDRMTQH-ALVAAEWALADAGLEP-EKQD
PdmB            RFDASRYPSPFGGEVPGFDAAERVPGRLIPQTDHWTHL-ALAATDLALADAGVVP-AELP
JadB            RFDPTGYPARLAGEVPGFAAEEHLPSRLLPQTDRMTRL-ALVAADWALADAGVRP-EEQD
UrdB            RFDPSRYPVQLAGEIEGFDAKGHLPGRLLPQTDRMTQL-ALVAADWAFEDAAVRP-GDLP
AlpB            RFDPTGYPARLAGQIDGFEADRLLPSRLLPQTDRVTRL-ALVAADWALADAGADP-AQLP
Aur1E           RFDASAYPSRLAGEIHGFEPKEHLPGRLVPQTDRVTQL-ALVAADCAFADAGIEP-GTID
Gris-ORF2       RFDASGYPARLAGEIRGFSAADHLPGRLVAQTDRVTQL-ALVAADRAFRDAGVAP-GDLP
LanB            RFDPTQYPARLAGEIRGFDARDHLPGRLIPQTDRMTQL-ALVATDSAFEDAGVKP-GDIP
PgaB            HFDPSSYPARLAGEIRGFEAKDHLPSRLIPQTDRMTQL-ALVAADCAFEDAGVEL-GNIP
SimA2           RFDPSQYPSRLAGEVPGFVAEDHLPSRLLPQTDHMTRL-ALVSADWALQDAGIRP-EELP
Dau-ORFB        RFADDGRLGRLAGEVSDFVPEDHLPKRLLVQTDPMTQMTALAAAEWALREAGCAP-SS--
DpsB            RFTGDGRLGRLAGEVSDFVPEDHLPKRLLAQTDPMTQY-ALAAAEWALRESGCSP-SS--
ActI-ORF2       RFDVSRYPATLAGQIDDFHAPDHIPGRLLPQTDPSTRL-ALTAADWALQDAKADP-ESLT
Qin-ORF18       RFDASRYAASLAGQIDDFDAAEHLNSRLLPQTDPSTRL-ALVAADWALTDADVSP-DTLP
Gra-ORF2        RFDASRYPSRLAGQIDDFEASEHLPSRLLPQTDVSTRY-ALAAADWALADAGVGPESGLD
Med-ORF2        RFDASRYPSRLAGQILGFDPAEHLPNRLLPQTDVSTRL-ALVAAEQALADGGVDP-AELV
AlnM            DYDASRYPSRLVGRIDGFEAAEHIPGRLLPQTDRVTRL-ALVAGAEALADADANP-AELA
FrnM            EYDASGHPGGLVGRVPDFDAARHLPGRLLPQTDRVTRL-ALVAADEALKDAAVDP-ARLP
Aur2B           RFDATQYPAKLAGEVPGFDAAEHVPSRLLPQTDHMTRL-ALTAADEALADAGVDP-AELP
MtmK            RFDPGRYPARLAGEIRDFVDADHVPGRLLPQTDRVTRL-SLAVAREAVEDAGVDL-ERLP
OxyB            RFDPSGYTSSLAGEIADFDPAR-LPNRLLPQTDLMTRL-ALVAAEEALDDAGADP-RTMP
                 :        . *.:  *     :  *:: ***  *:  :*.    *. :.
```

```
ElmL            G------FSTGVITASAAGGFEFGQRELQKLWGSGPGEVSAYQSFAWFYAVNTGQISIRH
TcmL            E------YGLGVLTAAGAGGFEFGQREMQKLWGTGPERVSAYQSFAWFYAVNTGQISIRH
PdmB            E------YEMAVVTASSSGGVEFGQREIQALWRDGPRHVGAYQSIAWFYAATTGQISIRH
JadB            D------FDMGVVTASASGGFEFGQGELQKLWSQGSQYVSAYQSFAWFYAVNSGQISIRN
UrdB            E------FEMGVITASSSGGFEFGQRELQALWSRGSRYVSAYQSFAWFYAVNSGQISIRN
AlpB            E------FDMGVITASAAGGFEFGQGELQALWSQGSQYVSAYQSFAWFYAVNSGQISIRN
Aur1E           P------YAMGVVTAAGAGGFEFAENELRKLWSEGAKHVSAYQSFAWFYAVNSGQISIRN
Gris-ORF2       A------NGMGVVTAAGSGGFEFGERELRKLWSLGANHVSAYQSFAWFPTANTGQIAIRS
LanB            E------YDMGVVTASTAGGFEFGQNELQALWSKGSQHVSAYQSFAWFYAVNSGQISIRN
PgaB            A------YDMGVVTASTSGGFEFGQNELKKLWSQGSRYVSAYQSFAWFYAVNSGQISIRN
SimA2           E------YAAGVVTASSAGGFEFGQNELRALWSKGSQYVSAYQSFAWFYAVNTGQISIRH
Dau-ORFB        P------LEAGVITASASGGFASGQRELQNLWSKGPAHVSAYMSFAWFYAVNTGQIAIRH
DpsB            P------LEAGVITASASGGFAFGQRELQNLWSKGPAHVSAYMSFAWFYAVNTGQIAIRH
ActI-ORF2       D------YDMGVVTANACGGFDFTHREFRKLWSEGPKSVSVYESFAWFYAVNTGQISIRH
Qin-ORF18       D------YDMGVVTSNALGGFDFTHREFDKLWNKGPDFVSVYESFAWFYAVNTGQISIRH
Gra-ORF2        D------YDLGVVTSTAQGGFDFTHREFHKLWSQGPAYVSVYESFAWFYAVNTGQISIRN
Med-ORF2        D------FDLGVVITSNASGGFAFTHREFANLWSKGPEYVSVYESFAWFYAVNTGQVSIRH
AlnM            EQDGYGEYGCGVVTSNATGGFEFTHREIRKLWTQGPQQVSVYESFAWFYAVNTGQLSIRH
FrnM            ------EYGASAVTSNATGGFEFTHREIRKLWTEGPARVSVYESFAWFYAVNTGQISIRH
Aur2B           D------FSAGVITASSAGGFEFGQKELQALWSQGGQYVSAYQSFAWFYAVNTGQISIKN
MtmK            R------YAAGVSTASSAGGFEFGQRELQALWSKGGQYVSAYQSFAWFYAVNTGQISIGY
OxyB            D------FAAGVVTAASAGGFDFGQRELEALWSKGGAHVSAYQSFAWFYPVNSGQISIRH
                          .. *:   **.   . *:  **  *   *..* *:*** ...:**::*

ElmL            GLRGHSSVAVAEQAGGLDAVAQATRLVDHGTLRVAVAGGFEAPVSPWGLVAQIPSGLLST
TcmL            GMRGHSSVFVTEQAGGLDAAAHAARLLRKGTLNTALTGGCEASLCPWGLVAQIPSGFLSE
PdmB            GMRGPCGVVVAEQAGALESFAQARRYLADG-ARVVVSGGTDAPFSPYGLTCQLGSGRLST
JadB            GMKGPSGVVVSDQAGGLDAVAQARRQIRKG-TRLIVSGGVDASLCPWGWVAHVASDRLST
UrdB            GMRGPSGVVVSDQAGGLDAVAQARRQIRKG-TRLVMSGAVDASICPWGWVAQMASNRLST
AlpB            GMKGPAGVVVSEGAGGLDAVAQARRQIRKG-TSLIVTGAVDASLCPWGWVAQLAGGRLST
Aur1E           GLRGPAGVVISDQAGGLDALAQARRQLRKG-SKLIATGGFDAPICSLGWASHLHGGLMST
Gris-ORF2       DSRGPNGVVVGDQAGGLDALGQARRLIRRG-TGLVAAGGFEASLTPLGWTARLSTGLMST
LanB            GMRGHSAVVVSDQAGGLDAIAQARRQIRKG-SKLICSGGVDASICPWGWVAQLANGRVST
PgaB            GMKGPSGVVVSDHAGGLDAIAQARRQIRKG-SKLIFSGGFDASICPWGWAAQIAGGRLSH
SimA2           GLRGPSGVVVSDQAGGLDALAQARRQIRRG-SQVIVSGGIDASICSWGWGAQLTSGRMST
Dau-ORFB        DLRGPVGVVVAEQAGGLDALAHARRKVRGG-AELIVSGAMDSSLCPYGMAAQVRSGRLSG
DpsB            DLRGPVGVVVAEQAGGLDALAHARRKVRGG-AELIVSGAVDSSLCPYGMAAQVKSGRLSG
ActI-ORF2       GMRGPSSALVAEQAGGLDALGHARRTIRRG-TPLVVSGGVDSALDPWGWVSQIASGRIST
Qin-ORF18       KLRGPSAALVGEQAGGLDALGHARRTIRRG-TPLVVAGGVDSALDPWGYVSQLAGGRVTT
Gra-ORF2        TMRGPSAALVGEQAGGLDAIGHARRTVRRG-PGWCSAVASTRRSTRGASSSQLSGGLVST
Med-ORF2        NVRGPGAALVAEQAGGLDALGHARRSLRLG-TPLVVTGGVDSALDPWGWASHLSSGLISD
AlnM            KLRGPSGVLVSEQAGGLDAIGQSRRTLRQG-VKLSLTGGMDSSLDPWGLVSHLASGRLSR
FrnM            GMRGPGAVVVADQAGGLDALGQARRVLRKG-GVLAVSGGVESALDPWGLAAHASSGTLSR
Aur2B           GLRGPSGVLVTEQAGGLDAVAQARRQLRKG-LRLVVTGGVDSSLCPWGWAAHLAGGELST
MtmK            DLRGPSGVLVTEQAGGLDAVAQARRLLRRG-SELMVTGGVDSALCPWGWTAHLAGGRLST
OxyB            DMRGPGGALVAEQAGGLDAVAKARRHVRDG-TPLMLTGGVDGSLCPWGWLCMTRSGALTT
                  :*   .. : : **.*:: .:: * :  *       : .        .  .    . ::

ElmL            VADPARAYLPFDPDASGWVPGEGGAALVVERAADARARGADHVYGRIAGHASTFDPRPGL
TcmL            ATDPHDAYLPFDARAAGYVPGEGGAMLVAERADSARERDAATVYGRIAGHASTFDARPGT
PdmB            GADPARAYLPFDAAANGFVPGEGGAILIIEQAATAQDR----SYGRIAGYAATFDPPPGS
JadB            SEEPARGYLPFDREAQGHVPGEGGAILVMEAAEAARERGAR-IYGEIAGYGSTFDPRPGS
UrdB            SRDPERAYLPFDDAAGGHVPGEGGALLVLEELEQARARGARQIYGVIAGYGSTLDPRPGS
AlpB            SDEPDHAYLPFDRDARGFVPGEGGAILIAEDAAAARTRGAR-PYGEIAGYGATIDPRPGS
Aur1E           SDEPERAYLPFDDAAAGYVPGEGGAMLILEDEDSARDRGARTVYGEFAGYGATLDPKPGS
Gris-ORF2       SDDPDRAYLPFHPAARGYVPGEGGALLILEDERSALARGAATAHAELAGYSATLDPRPGS
LanB            SENTERAYLPFDADASGYVPGEGGALLILEDAEAARQRGAENVYGEIAGYGSTFDPRPGS
PgaB            SDRPERAYLPFDRAANGYVPGEGGALLILEDERTARERGAEKIYGEVAGYGSTFDPRPGS
SimA2           DDDPATAYRPFDGAAAGHVPGEGGALLVLEEAGAAAARGAR-VYGRIAGYGTTFDPKPGS
Dau-ORFB        SDDPTAGYLPFDRRAAGHVPGEGGAILAVEDAERVAERGGK-VYGSIAGT-ASFDPPPGS
DpsB            SDNPTAGYLPFDRRAAGHVPGEGGAILTVEDAERAAERGAK-VYGSIAGYGASFDPPPGS
ActI-ORF2       ATDPDRAYLPFDERAAGYVPGEGGAILVLEDSAAAEARGRHDAYGELAGCASTFDPAPGS
Qin-ORF18       ATDPARAYLPFSADASGYVPGEGGAILVAEDEASARERGVQQVYGELAGYAATFDPAPGT
```

```
Gra-ORF2        VADPERAYLPFDVDASGYVPGEGGAVLIVEDADSARARGAERIY---VRSPLRRDPAPGS
Med-ORF2        SDDPDRAYLPFDARARGHVPGEGGAFLVMEDEQGALRRGAGQVYGELAGYAATFDPHPDS
AlnM            SDDPATAYLPFDTRAAGQVPGEGGAMLVLEEETAARARGA-RVYGEIAGYAATFSPRPGS
FrnM            SGDPATAYLPFDRRALGTVVGEGGALLTLETPRHAEERDAPRIYGELAGYAATFDPPAGS
Aur2B           SEDPARAFRPFSAEADGHVAGEGGALLVLEDAAAARERGA-EIYGTVAGYASTFDPAPGS
MtmK            GDDPARAYLPFSADADGEVVGEGGALLVLERAAAAPSRGA-RVYGVFAGYAATFDPPPGR
OxyB            RTDPRRAYLPFSPDASGYVPGEGGALLVLEDPRAAAERGAPQVYGRIAGYAATFDPRPGS
                   .  .: **   * * * ***** *  *    .  *     :   .      .. ..

ElmL            GRPPALARAVRTALDRARTHPGDIDVVFADAAGLPEQDAAEVAALTEVFGSRKVPVTAPK
TcmL            GRPTGPARAIRLALEEARVAPEDVDVVYADAAGVPALDRAEAEALAEVFGPGAVPVTAPK
PdmB            GRPPTLERAVRAALDDARLTPADVDVVFADAAGVPDLDRAEADAIGAVFGPRGVPVTAPK
JadB            GREPGLRKAIELALADAGAAPGDIDVVFADAAAVPELDRVEAEALNAVFGTGAVPVTAPK
UrdB            GRPAGLRKAIELALADAGAAPEEIDVVFADAAAVPELDRIEAEALTQVFGARAVPVTAPK
AlpB            GREPGLRKAIETALADARLSAADIDVVFADGAGDPAGDRIEADAISTVFGDRGVPVTVPK
Aur1E           GREPGLRRAIDVALTDAACHPAEVDVVFADGAATPRLDREEAEAITAVFGPRAVPVTVPK
Gris-ORF2       GREPGLRRAIELALADAGVAPAEVDVVFADAAGSPEPDAVEVAAVAGVFGPRAVPVTAPK
LanB            GREPGLRRAIELALADADAEPSDIGVVFADAAGTPELDRVEAEALIEVFGAAGVPVTAPK
PgaB            DREPGLRHAIEVALADAGVTAADVDVVFADAAGSPDLDRQEADAITAVFGPSGVPVTAPK
SimA2           GREPGLRRAIELALTDAGLAPADIDVVFADAAAVPDLDRIEARALVEVFGPRGLPVTAPK
Dau-ORFB        GRPSALARAVETALADAGLDRSDIAVVFADGAAVGELDVAEAEALASVFGPHRVPVTVPK
DpsB            GRPSALARAVETALADAGLDGSDIAVVFADGAAVPELDAAEAEALASVFGPRRVPVTVPK
ActI-ORF2       GRPAGLERAIRLALNDAGTGPEDVDVVFADGAGVPELDAAEARAIGRVFGREGVPVTVPK
Qin-ORF18       GRPPALRRAAEAALADAGLAPGDIDVVFADGAGVPELDAAEAAAISGLFGAGAVPVTAPK
Gra-ORF2        GRPPALGRAAELALAEAGLTPADISVVFADGAGVPELDRAEADTLARLFGPRGVPVTAPK
Med-ORF2        GRPPALRRAAELAIADAGLEPSDIGVVFADAAGSADLDRTEAEAIAAVFGPRGVPVAAPK
AlnM            GRPPGLERAARLALADSGLGVEDVDVVFADAAGLPTADDEEAAALRALFGPYGVPVTAPK
FrnM            GRPPGLERAARLALADAGLAPGDVDVVFADAAGLPAADAAEAAALRALFGPGGVPVSVPK
Aur2B           GGAPRLQAAAELALRDAGLTPADVDVVFADAAGRRDADRAEARALTALFGEYGVPVTAPK
MtmK            GGTPQLARAAELALVDAGMTPDQVDVVFADAAGERAADRAEADALTRVFGARGVPVTAPK
OxyB            GREPGLRRALRLALDDAGIGPADVDVVFADAMGVPALDAVETQVLAAEFGPRGVPVTAPK
                .  .    *   *:  :     :: **:**. .    *  *. .:    **   :**:.**

ElmL            TMTGRLYSGGGALDTATALLALRDGVVPPTVGTRTAPP--ELDLVLGAPR-DLPLRNALV
TcmL            TMTGRLYAGGAALDVATALLSIRDCVVPPTVGTGAPAPGLGIDLVLHQPR-ELRVDTALV
PdmB            SLTGRLYAGGPALDAATALLAMHDSVIPPTAGGADVPPGYALDLVGAEPR-PARLRTALI
JadB            TMTGRLYSGAAPLDLAAAFLAMDEGVIPPTVN-VEPDAAYGLDLVVGGPR-TAEVNTALV
UrdB            TMTGRLYSGAAPLDLAAAFLAMRDGVVPPSVG-VSPSPDHDLDLVVHQER-AMTVRSALV
AlpB            TMTGRLYSGGAPLDLAAAFLALRDGVIPPTVH-IDPCADYPLDLVLGEPR-PAPLRTALI
Aur1E           TMTGRINSGGAPIDVVSAVLSMREGLIPPTTN-VELSDAYDLDLVAVRPR-TASVRTALV
Gris-ORF2       TMIGRLQAGGAPVDVVTAVLAIREGLIPPTAD-AESATARELDLVVGRPR-TASVGTALV
LanB            TMTGRLYSGAAPVDVVTAVLAMREGLIPPTVN-VSLSPEYDIDLVTAQPR-TARVRNALV
PgaB            TMIGRLYSGAAPVDVVAAVLAIREGLIPPTTN-VELSPDYDLDLVTGQPR-TASVRTALV
SimA2           SMTGRLNSGAGSLDVATALLAIRDGVIPPTIN-VTAQDDYELDLVTAQAR-SARLRSALV
Dau-ORFB        TLTGRLYSGAGPLDVATGLLALRDEVVPATGH-VHPDPDLPLDVVTGRPRAMADARAALV
DpsB            TLTGRLYSGAGPLDVATALLALRDEVVPATAH-VDPDPDLPLDVVTGRPRSLADARAALL
ActI-ORF2       TTTGRLYSGGGPLDVVTALMSLREGVIAPTAGVTSVPREYGIDLVLGEPR-STAPRTALV
Qin-ORF18       ALTGRLYSGGGPLDVATALLSIRDGVIPPTPTGAPVPEDYGLDLVQGAPR-DQAVRTALV
Gra-ORF2        ALTGRLCAGGGPADLAAALLALRDQVIPATGRHRAVPDAYALDLVTGRPR-EAALSAALV
Med-ORF2        ALTGRLLAGGGPLDVVAASVRLRDGLLPAVPYEGETPDAYGIDLVRGTPR-PTSARAALV
AlnM            TLTGRLFGGGAPLDVAAALLALRDGVIPPTAGIDRPVPEHRLDLVRGTPR-HTPLRTALV
FrnM            TQTGRLASGGPALDVAAALLALRDGLVPPAVHLDEVDPAYGLDLVRDTPR-ALPLRTALV
Aur2B           TMTGRLAAGGASLDLAAALLSLRDQVIPPTVNTGPAAEDCPVDLVSEVRR-PERLRTALV
MtmK            TMTGRLSAGGASLDLAAALLALRDQVVPPTVNVTEPADDCPVDLVTGRPR-PLPLRAALV
OxyB            TMTGRLLAGGASLDLAAALLSLRDQVVPPTVHVDGGEIPDSLDLVTGAPR-PARLRHALV
                :  **: .*. . * .:. : : : ::..            :*:*    *       **:

ElmL            LARGTGGFNSAVVVARV--------
TcmL            VARGMGGFNSALVVRRHG-------
PdmB            IARGYGGFNAALVLRGPNT------
JadB            IARGHGGFNSAMVVRSAN-------
UrdB            IARGHGGFNSAVVVRAVG-------
AlpB            LARGHGGFNSAMAVRAV--------
Aur1E           LARGRGGFNSAVVVRAVD-------
```

```
Gris-ORF2       LARGHGGFNSAVVLRAVD-------
LanB            VARGYGGFNSAMVVRGTDR------
PgaB            LARGVGGFNSAVVVRAVD-------
SimA2           VARGHGGFNSALVVQDAA-------
Dau-ORFB        VARGHGGFNSALVVRGAA-------
DpsB            VARGYGGFNSALVVRGAA-------
ActI-ORF2       LARGRWGFNSAAVLRRFAPTP----
Qin-ORF18       LARGRHGFNSAVVVRA---------
Gra-ORF2        LARGRHGFNSAVVVTLRGSDHRRPT
Med-ORF2        LARGRWGFNSAVVVKAADRG-----
AlnM            LARGHGGFNAAVVVR---APEAA--
FrnM            LARGHGGFNAAVVVRGRRRPRTA--
Aur2B           LARGRGGFNAAMVVRAAH-------
MtmK            LARGRGGFNAAAVVRALS-------
OxyB            LARGHGGFNSAMVVSGRD-------
                :***  ***:* .:
```

Fig. S11. Comparison of the amino acid sequence of KSβ (Aur1E, Aur2B) *from S. lavendulae* subsp. *lavendulae* CCM 3239 with selected KSβ proteins from the basic groups of aromatic polyketides. Protein sequences (and corresponding accession numbers) are as follows: elloramycin ElmL (CAP12601), tetracenomycin TcmL (AAA67516), pradimicin PdmB (ABM21748), jadomycin JadB (AAB36563), urdamycin UrdB (CAA60570), kinamycin AlpB (AAR30151), auricin Aur1E (AAX57192), griseusin Gris-ORF2 (CAA54859), landomycin LanB (AAD13537), gaudimycin PgaB (AAK57526), simocyclinone SimA2 (AAK06785), daunorubicin Dau-ORFB (AAA87619), doxorubicin DpsB (AAA65207), actinorhodin ActI-ORF2 (CAC44201), qinimycin Qin-ORF18 (WP_058047373), granaticin Gra-ORF2 (CAA09654), medermycin Med-ORF2 (BAC79045), alnumycin AlnM (ACI88862), frenolicin FrnM (AAC18108), auricin Aur2B (AIE41911), mithramycin MtmK (CAA61990), oxytetracycline OxyB (AAZ78326).

```
CLUSTAL X (1.81) multiple sequence alignment
PgaA            ----VSRRVVITGVGVLAPGGIGAKNFWSLLSEGRTATRGITFFDPSSFRSRVAAEADFD
LanA            ----MGRRVVVTGIGVLAPGGIGAENFWSLLSEGRTATRGITFFDPSSFRSQIAAEADFD
AlpA            ---LSRRRVVITGIEVIAPGGVGRENFWNLLSSGRTATRGITFFDPAPFRSRVAAEADFD
JadA            ---VTARRVVITGIEVLAPGGTGSKAFWNLLSEGRTATRGITFFDPTPFRSRVAAEIDFD
UrdA            VSGAHSRRVVITGIGVTAPGGVGSKNFWSLLSDGRTATRRISFFDPSPFRSQVAAEADFD
SimA1           ----VRRRVVITGVGVMAPGGIGVKNFWSLLSEGRTATRGITFFDPAPFRSRVAAEIDFF
Aur1D           ----MTRRVVITGIGVLAPGGIGTTAFWNLLSSGRTATRGITLFDPAPFRSRVAAEVDFH
                      ****:**: * **** *   **.***.****** *::***:.***::*** **

PgaA            PEMHGLTPQEIRRMDRAAQFAVVTAREALADSGLDLAGFDPHRTGVTIGSAVGATTGLDD
LanA            AERSGLSPQEIRRMDRAAQFGVVTAREALADSGLDQAGLDPYRTGVTIGSAVGATMGLDE
AlpA            PFAHGLSPQEVRRLDRAAQFAVVASRGAVADSGLDVAALDPHRVGVTVGSAVGATMGLDE
JadA            PEAHGLSPQEIRRMDRAAQFAVVAAR-AVADSGIDLAAHDPYRVGVTVGSAVGATMGLDE
UrdA            AELLGLSPQEIRRMDRAAQFAVVTAREAVADSGLEFASLDPHRTGVTVGSAVGATMGLDQ
SimA1           PEKHGLGPQKIRRMDRAAQLAVIATREAVADSGLDLDATDPHRRGVTVGSAVGATTGLDQ
Aur1D           PERHGLTSLEVRRMDRAAQFGVVATREALADSGLDLAAFDPYRIGIAMGTGVGAISGLDT
                .   ** . ::**:*****:.*:::* *:****::  . **:* *:::*:.***  ***

PgaA            EYRVVSDGGRLDLVDHTYAPPHLYNYFVPSSFSAEVAWAVGAEGPATVVSTGCTSGLDSV
LanA            EYRVVSDGGRLDLVDHRYTPQHLYNHFVPSSFSAEVAWAVGAEGPNTVVSTGCTSGIDSV
AlpA            EYRVVSDGGRLEAVDHTYAVPHLYDYMVPSSFAAEVAWAVGAEGPSTVVSTGCTSGIDSV
JadA            EYRVVSDGGRLDLVDHAYAVPHLYDYMVPSSFSAEVAWAVGAEGPNTVVSTGCTSGLDSV
UrdA            EYRTVSDSGRLDLVDHEYAVPHLYNYLVPSSFAAEVAWAVGAEGPATVVSTGCTSGLDAV
SimA1           EYRVVSDDGRLGLVDHRYAPPHLYGHFVPSSFAAEVAWASGSEGPSTVVSTGCTSGLDSL
Aur1D           AYRVASDEGRIAQVDHMYAPGHLYDHFVPSSFATEVAWAVGAEGPSTVVSTGCTSGIDSV
                 **..** **:  *** *:  ***.::*****::***** *:*** **********:*::

PgaA            GHAT-ELIREGTADVMITGATDAPISPITMACFDAIKATTPRNDDAAHASRPFDGTRNGF
LanA            GQAV-ELIREGSVDVMIAGATDAPISPITMACFDAIKATTPRNDEPEHASRPFDGTRNGF
AlpA            GYAV-ELVREGSADVVIAGSSDAPISPITMACFDAIKATTPRHDEPECASRPFDKTRNGF
```

```
JadA            GYARGELIREGSADVMIAGSSDAPISPITMACFDAIKATTNRYDDPAHASRPFDGTRNGF
UrdA            GYAT-ELIREGSADVMVAGAADAPISPITVACFDAIKATTPRNEDPEHASRPFDGTRNGF
SimA1           GHAV-ELIREGSADVMIAGATDAPISPITMACFDAIKATTPRNDDPAHASRPFDRSRNGF
Aur1D           AHAV-DLIREGSADIMITGATDAPISPLTLACFDAIKATTALNEEPETASRPFDATRKGF
                . *  :*:***:.*::::*::******:*:**********   ::.  ****** :*:**

PgaA            VLGEGSAVFVLEELESAKKRGAHIYAEIAGYASRCNAFHMTGLRPDGREMSEAIDVALGE
LanA            VLGEGSAVFVLEELTAARARGAHIYAEIAGYASRCNAFHMTGLRPDGREMGEAIRVALDE
AlpA            VLGEGCAFFVLEELDSARKRGAHIYAEIAGYSTRSNAYHMTGLRPDGAEMAEAIDLALAE
JadA            VLGEGAAVFVLEELESARARGAHIYAEIAGYATRSNAYHMTGLRPDGAEMAEAIRVALDE
UrdA            VLGEGSAVFVLEELDSALRRGAHVYAEIAGYATRSNAFHMTGLRPDGREMAEAIRIALDE
SimA1           VLGEGHAVLVLEELGAAQRRGARIYAEIGGYASRCNAFHMTGLRPDGREMAEAIRVALDE
Aur1D           VLGEGAAAFVLEELGAARRRGAHVYAEIAGYASRSSAFHMTGLRPDGREMAEAITVALDE
                ***** * :***** :*  ***::****.**::*..*:********* **.*** :** *

PgaA            ARMNPDRIDYINAHGSGTKQNDRHETAAFKLSLGDHAYRTPVSSIKSMVGHSLGAIGSIE
LanA            ARINPEAIDYINAHGSGTKQNDRHETAAFKLSLGEHAYRTPISSIKSMVGHSLGAIGSIE
AlpA            ARLNPQAIDYVNAHGSGTKQNDRHETAAFKRSLGEHAFRTPVSSIKSMVGHSLGAIGSIE
JadA            ARMNPTEIDYINAHGSGTKQNDRHETAAFKKSLGDHAYRTPVSSIKSMVGHSLGAIGSIE
UrdA            ARLNPEDIDYVNAHGSGTKQNDRHETAAFKRSLGDHAYAVPVSSIKSMVGHSLGAIGSIE
SimA1           ARLNPEDIDYINAHGSGTKQNDRHETAAFKRSLGDHAYRTPVSSIKSMVGHSLGAIGSIE
Aur1D           ARLDPGDLDYVNAHGSGTRQNDLHETAAYKLALGPHAHRTPVSSVKSMIGHSLGSVGSME
                **::*  :**:*******:*** *****:* :** **. .*:**:***:*****::**:*

PgaA            IAASVLAMKNHVVPPTANLHTPDPECDLDYVPLTAREQRTDTVLTVGSGFGGFQSAMVLA
LanA            IAASLLAMENHVVPPTANLHTPDPECDLDYVPLVAREHTTDTVLTVGSGFGGFQSAMVLA
AlpA            IAASALAMEYDVVPPTANLHTADPECDLDYVPLVARDQLIDAVLTVGSGFGGFQSAMVLA
JadA            IAASALAMEHNVVPPTGNLHTPDPECDLDYVR-SCREQLTDSVLTVGSGFGGFQSAMVLA
UrdA            IAASALAMEHGVVPPTANLHTPDPECDLDYVPRTARDWKTDAVLSVGSGFGGFQSAIVLA
SimA1           IAACVLAMAESVIPPTANLHDPEPECDLDYVPITAREARLDRVLSVGSGFGGFQSAMVLT
Aur1D           IAASVLAMEHHVVPPTANLRTPDPECDLDYVPLVARDHRTDAVLTVSSGFGGFQSAMVLA
                ***. ***   *:***.**: .:********   .*:   * **:*.*********:**:

PgaA            RPERNAA
LanA            RPERSAA
AlpA            SPERSLV
JadA            RPERKIA
UrdA            RPDRRTA
SimA1           RPERNTA
Aur1D           RPDRRTA
                 *:*  .
```

Fig S12. Comparison of the amino acid sequence of Aur1D *from S. lavendulae* subsp. *lavendulae* CCM 3239 with the corresponding KSα proteins from the angucycline BGCs. Protein sequences (and corresponding accession numbers) are as follows: PgaA (AAK57525), LanA (AAD13536), AlpA (CAJ87874), JadA (AAB36562), UrdA (caa60569), SimA1 (AAK06784, Aur1D (AAX57191). Amino acid residues highlighted in green correspond to the Cys-His-His catalytic triad (Keatinge-Clay et al. 2004), highlighted in yellow different residues in Aur1D that were conserved in all other KSs.

```
CLUSTAL X (1.81) multiple sequence alignment
AlpB            ---MTASVVVTGLGVTAPNGLGLKDYWAATLGGKHGIGRITRFDPTGYPARLAGQIDGFE
JadB            ---MSASVVVTGLGVAAPNGLGREDFWASTLGGKSGIGPLTRFDPTGYPARLAGEVPGFA
UrdB            VNTGAVEVAVTGLGVVAPNGLGTDAYWAATRKGTSGIARISRFDPSRYPVQLAGEIEGFD
SimA2           ---MTTSVVVTGLGVAAPNGLGTADYWAATREGRSGIGRVTRFDPSQYPSRLAGEVPGFV
PgaB            ---MSTRTVITGIGVATPNGLGVDEFWAATRVGKNAIARVTHFDPSSYPARLAGEIRGFE
Aur1E           ---MSARILVTGIGVAAPSGLGVEDFWSVTRIGKNAIGPVTRFDASAYPSRLAGEIHGFE
LanB            ---MTARVVITGIGIAAPNGFGVEDYWAATRVGKSAIGRITRFDPTQYPARLAGEIRGFD
                    :.   :**:*:.:*.*:*   :*: *  *  .*. :::**.: ** :***:: **
```

```
AlpB            ADRLLPSRLLPQTDRVTRLALVAADWALADAGADPAQLPEFDMGVITASAAGGFEFGQGE
JadB            AEEHLPSRLLPQTDRMTRLALVAADWALADAGVRPEEQDDFDMGVVTASASGGFEFGQGE
UrdB            AKGHLPGRLLPQTDRMTQLALVAADWAFEDAAVRPGDLPEFEMGVITASSSGGFEFGQRE
SimA2           AEDHLPSRLLPQTDHMTRLALVSADWALQDAGIRPEELPEYAAGVVTASSAGGFEFGQNE
PgaB            AKDHLPSRLIPQTDRMTQLALVAADCAFEDAGVELGNIPAYDMGVVTASTSGGFEFGQNE
Aur1E           PKEHLPGRLVPQTDRVTQLALVAADCAFADAGIEPGTIDPYAMGVVTAAGAGGFEFAENE
LanB            ARDHLPGRLIPQTDRMTQLALVATDSAFEDAGVKPGDIPEYDMGVVTASTAGGFEFGQNE
                .   **.**:****::*:****::* *: **.        :  **:**: :*****.: *

AlpB            LQALWSQGSQYVSAYQSFAWFYAVNSGQISIRNGMKGPAGVVVSEGAGGLDAVAQARRQI
JadB            LQKLWSQGSQYVSAYQSFAWFYAVNSGQISIRNGMKGPSGVVVSDQAGGLDAVAQARRQI
UrdB            LQALWSRGSRYVSAYQSFAWFYAVNSGQISIRNGMRGPSGVVVSDQAGGLDAVAQARRQI
SimA2           LRALWSKGSQYVSAYQSFAWFYAVNTGQISIRHGLRGPSGVVVSDQAGGLDALAQARRQI
PgaB            LKKLWSQGSRYVSAYQSFAWFYAVNSGQISIRNGMKGPSGVVVSDHAGGLDAIAQARRQI
Aur1E           LRKLWSEGAKHVSAYQSFAWFYAVNSGQISIRNGLRGPAGVVISDQAGGLDALAQARRQL
LanB            LQALWSKGSQHVSAYQSFAWFYAVNSGQISIRNGMRGHSAVVVSDQAGGLDAIAQARRQI
                *: ***.*:::**************:******:*::* :.**:*: ******:******:

AlpB            RKGTSLIVTGAVDASLCPWGWVAQLAGGRLSTSDEPDHAYLPFDRDARGFVPGEGGAILI
JadB            RKGTRLIVSGGVDASLCPWGWVAHVASDRLSTSEEPARGYLPFDREAQGHVPGEGGAILV
UrdB            RKGTRLVMSGAVDASICPWGWVAQMASNRLSTSRDPERAYLPFDDAAGGHVPGEGGALLV
SimA2           RRGSQVIVSGGIDASICSWGWGAQLTSGRMSTDDDPATAYRPFDGAAAGHVPGEGGALLV
PgaB            RKGSKLIFSGGFDASICPWGWAAQIAGGRLSHSDRPERAYLPFDRAANGYVPGEGGALLI
Aur1E           RKGSKLIATGGFDAPICSLGWASHLHGGLMSTSDEPERAYLPFDDAAAGYVPGEGGAMLI
LanB            RKGSKLICSGGVDASICPWGWVAQLANGRVSTSENTERAYLPFDADASGYVPGEGGALLI
                *:*: :: :*..**.:*. ** ::: .. :* .  .  .* ***  * *.*******:*:

AlpB            AEDAAAARTRGAR-PYGEIAGYGATIDPRPGSGREPGLRKAIETALADARLSAADIDVVF
JadB            MEAAEAARERGAR-IYGEIAGYGSTFDPRPGSGREPGLRKAIELALADAGAAPGDIDVVF
UrdB            LEELEQARARGARQIYGVIAGYGSTLDPRPGSGRPAGLRKAIELALADAGAAPEEIDVVF
SimA2           LEEAGAAAARGAR-VYGRIAGYGTTFDPKPGSGREPGLRRAIELALTDAGLAPADIDVVF
PgaB            LEDERTARERGAEKIYGEVAGYGSTFDPRPGSDREPGLRHAIEVALADAGVTAADVDVVF
Aur1E           LEDEDSARDRGARTVYGEFAGYGATLDPKPGSGREPGLRRAIDVALTDAACHPAEVDVVF
LanB            LEDAEAARQRGAENVYGEIAGYGSTFDPRPGSGREPGLRRAIELALADADAEPSDIGVVF
                 *    *  ***.  ** .****:*:**:***.* .***:**: **:**   . ::.***

AlpB            ADGAGDPAGDRIEADAISTVFGDRGVPVTVPKTMTGRLYSGGAPLDLAAAFLALRDGVIP
JadB            ADAAAVPELDRVEAEALNAVFGTGAVPVTAPKTMTGRLYSGAAPLDLAAAFLAMDEGVIP
UrdB            ADAAAVPELDRIEAEALTQVFGARAVPVTAPKTMTGRLYSGAAPLDLAAAFLAMRDGVVP
SimA2           ADAAAVPDLDRIEARALVEVFGPRGLPVTAPKSMTGRLNSGAGSLDVATALLAIRDGVIP
PgaB            ADAAGSPDLDRQEADAITAVFGPSGVPVTAPKTMIGRLYSGAAPVDVVAAVLAIREGLIP
Aur1E           ADGAATPRLDREEAEAITAVFGPRAVPVTVPKTMTGRINSGGAPIDVVSAVLSMREGLIP
LanB            ADAAGTPELDRVEAEALIEVFGAAGVPVTAPKTMTGRLYSGAAPVDVVTAVLAMREGLIP
                **.*. *  ** ** *:  ***  .:***.**:* **: **...:*:.:*.*:: :*::*

AlpB            PTVHIDPCADYPLDLVLGEPRPAPLRTALILARGHGGFNSAMAVRAV--
JadB            PTVNVEPDAAYGLDLVVGGPRTAEVNTALVIARGHGGFNSAMVVRSAN-
UrdB            PSVGVSPSPDHDLDLVVHQERAMTVRSALVIARGHGGFNSAVVVRAVG-
SimA2           PTINVTAQDDYELDLVTAQARSARLRSALVVARGHGGFNSALVVQDAA-
PgaB            PTTNVELSPDYDLDLVTGQPRTASVRTALVLARGVGGFNSAVVVRAVD-
Aur1E           PTTNVELSDAYDLDLVAVRPRTASVRTALVLARGRGGFNSAVVVRAVD-
LanB            PTVNVSLSPEYDIDLVTAQPRTARVRNALVVARGYGGFNSAMVVRGTDR
                *:  :     : :***    *.  :..**::*** ******:.*: .
```

Fig S13. Comparison of the amino acid sequence of Aur1E *from S. lavendulae* subsp. *lavendulae* CCM 3239 with the corresponding KSβ proteins from the angucycline BGCs. Protein sequences (and corresponding accession numbers) are as follows: AlpB (CAJ87873), JadB (AAB36563), UrdB (CAA60570), SimA2 (AAK06785), PgaB (AAK57526), Aur1E (AAX57192), LanB (AAD13537). Amino acid residues highlighted in green correspond to the altered catalytic triad in CLF (Keatinge-Clay et al. 2004), highlighted in yellow different residues in Aur1E that were conserved in all other KSs.
